# Metabolic Reprogramming-Driven Cardiovascular Immune Damage: From Glyco-Lipotoxicity and Epigenetic Memory to Multidimensional Cross-Organ Communication Networks

**DOI:** 10.3390/ijms27125526

**Published:** 2026-06-18

**Authors:** Zijin Sun, Yongchao Liu, Kai Wang, Haojia Zhang, Rui Zhou, Wei Shao

**Affiliations:** 1 School of Traditional Chinese Medicine, Beijing University of Chinese Medicine, Beijing 102488, China; 2 School of Chinese Materia Medica, Beijing University of Chinese Medicine, Beijing 102488, China

**Keywords:** metabolic reprogramming, glyco-lipotoxicity, trained immunity, NLRP3 inflammasome, epigenetic remodelling, cross-organ communication

## Abstract

Cardiovascular disease (CVD) remains the leading cause of mortality worldwide, and residual inflammatory risk persists despite optimal lipid and glucose control. Emerging evidence indicates that metabolic reprogramming within immune cells constitutes a central driver of cardiovascular immune injury. In this review, we propose a unifying framework in which glyco-lipotoxicity acts as a primary metabolic trigger, inducing mitochondrial dysfunction, oxidative stress, and activation of the NLRP3 inflammasome and cGAS–STING pathways. Hyperglycaemia and dyslipidaemia reshape intracellular metabolic circuits, enhancing glycolysis and disrupting oxidative phosphorylation, thereby promoting sustained pro-inflammatory phenotypes. Crucially, metabolic intermediates function as cofactors for epigenetic remodelling. This establishes trained immunity in both circulating innate immune cells and haematopoietic stem/progenitor cells, which serves as the cellular basis for persistent metabolic memory. This persistent immunometabolic imprint amplifies sterile inflammation and accelerates vascular and myocardial remodelling. Furthermore, these processes are systemically propagated through cross-organ communication networks, including the heart–adipose, gut–heart, and cardio-hematopoietic axes, forming a multidimensional inflammatory amplification loop. We also summarise emerging therapeutic strategies targeting the metabolic–epigenetic axis, aiming to reverse maladaptive trained immunity and mitigate residual CVD risk. By integrating immunometabolism, epigenetic regulation, and organ crosstalk, this review highlights metabolic reprogramming as a pivotal mechanistic nexus and potential precision target for cardiovascular protection.

## 1. Introduction

### 1.1. Epidemiological Paradox: The Persistent Risk of Residual Inflammation

Current cardiovascular disease (CVD) prevention and control strategies primarily focus on managing traditional risk factors such as low-density lipoprotein cholesterol (LDL-C) and blood pressure. However, epidemiological data reveal a sobering reality: even among patients receiving intensive lipid-lowering therapy with statins, where LDL-C levels have been reduced to guideline-recommended targets (e.g., <70 mg/dL), the incidence of major adverse cardiovascular events (MACE) remains persistently high [1,2,3]. This phenomenon, termed ‘residual cardiovascular risk,’ indicates that relying solely on lipid-centric treatment strategies is insufficient to fully halt the progression of atherosclerosis [4]. Large-scale clinical cohort studies further confirm that, in coronary heart disease patients on statin therapy, residual cholesterol risk alone shows no significant association with MACE. Conversely, residual inflammatory risk (RIR)—typically signified by elevated high-sensitivity C-reactive protein (hsCRP)—emerged as an independent and potent predictor of all-cause mortality, myocardial infarction, and stroke risk [5,6]. For instance, in the CLEAR-Outcomes trial, hsCRP demonstrated superior predictive value for future cardiovascular events and mortality compared to LDL-C in high-risk patients intolerant to statins [7].

Beyond patients with established CVD, a paradoxical phenomenon also exists within the general population. Conventional screening typically focuses on the four standard modifiable risk factors (SMuRFs): hypertension, hyperlipidaemia, diabetes mellitus, and smoking. However, studies reveal that over a 30-year follow-up, women lacking these traditional risk factors but presenting with elevated baseline hsCRP levels (termed ‘SMuRF-less but inflamed’) exhibited significantly increased risks of coronary heart disease and ischaemic stroke [8]. Each quintile increase in hsCRP levels was associated with a 21% rise in coronary heart disease risk [8]. Furthermore, long-term follow-up data indicate that baseline hsCRP levels not only independently predict long-term cardiovascular events in hypertensive patients but also reflect age-related vascular dysfunction and atherogenic processes [9,10]. This evidence suggests systemic inflammation is not merely a concomitant of CVD but an independent driver distinct from lipid profiles, constituting the core of residual risk [7,8].

The pathophysiological basis of residual inflammation risk is closely associated with “meta-inflammation”—a chronic, low-grade, sterile inflammatory state triggered by nutritional excess and metabolic dysfunction [11]. Complex interactions between metabolic substrates and the immune system form a multidimensional pathogenic network. Research indicates a synergistic effect between hyperlipidaemia and inflammation in increasing cardiovascular event risk: when LDL-C, lipoprotein(a) [Lp(a)], and hsCRP are simultaneously elevated, patient prognosis significantly deteriorates [12,13]. Notably, elevated levels of triglyceride-rich lipoprotein remnants (remnant cholesterol) are independently associated with cardiovascular events and positively correlated with systemic inflammation, with inflammation partially mediating the pathogenic effects of remnant cholesterol [14]. Moreover, oxidised phospholipids (OxPLs), as metabolic by-products, can accumulate on apolipoprotein B and induce persistent chronic inflammation [15]. These findings indicate that metabolic reprogramming not only leads to lipid deposition but also sustains microenvironmental inflammation within the vascular wall by activating the immune system, thereby driving immune injury and plaque instability [11,16]. However, a significant knowledge gap persists in the current understanding of RIR: while the clinical importance of inflammation is well-recognised, we still lack a cohesive framework that explains how transient or chronic metabolic disturbances are “encoded” into a long-lasting inflammatory memory that fuels cardiovascular progression even after traditional risk factors are controlled. Most existing literature focuses on isolated pathways or single biomarkers, failing to provide a multidimensional view of the “metabolic-epigenetic-systemic” axis. Therefore, the primary objective of this review is to bridge this gap by constructing a comprehensive pathophysiological framework. We aim to elucidate how glucolipotoxicity acts as a metabolic trigger to initiate intracellular reprogramming and “trained immunity,” and how these cellular events are amplified through systemic cross-organ communication, ultimately driving accelerated cardiovascular remodelling [17].

### 1.2. Conceptual Framework of Immunometabolism

The core definition of immunometabolism lies in the strict coupling between the functional phenotype of immune cells and their intracellular metabolic pathways. Accordingly, immunometabolic reprogramming is defined as the dynamic alteration of these intracellular metabolic circuits (e.g., shifts between glycolysis and oxidative phosphorylation) to adapt to microenvironmental cues and support specific immune effector functions. Research indicates that the activation, differentiation, and effector functions (phagocytosis, migration, cytokine secretion, etc.) of immune cells (such as macrophages and T cells) are not merely passive energy-consuming processes. Instead, they are finely regulated by specific metabolic programmes, including glycolysis, oxidative phosphorylation (OXPHOS), fatty acid oxidation (FAO), and amino acid metabolism [18,19,20]. This metabolic reprogramming not only supplies cells with energy (ATP) but, more importantly, provides intermediates required for biosynthesis, directly shaping the plasticity of immune cells [21,22]. For instance, pro-inflammatory M1 macrophages typically undergo metabolic switching, relying on aerobic glycolysis while suppressing the tricarboxylic acid (TCA) cycle to rapidly generate reactive oxygen species (ROS) and inflammatory cytokines. In contrast, anti-inflammatory/reparative M2 macrophages primarily depend on the intact TCA cycle, OXPHOS, and fatty acid oxidation to sustain their functions [19,20,23]. Similarly, CD4+ effector T cells (Teff) rely on glycolysis and glutamine catabolism, whereas regulatory T cells (Treg) depend more heavily on fatty acid oxidation (FAO). This metabolic preference determines the differentiation fate of T cells [21].

At the molecular mechanism level, metabolic reprogramming in immune cells is regulated by intricate signalling networks. Key metabolic checkpoints such as mTOR, AMPK, and HIF-1α play pivotal roles in this process, sensing nutritional status and guiding immune cell polarisation [24,25]. Beyond core glycolytic and lipid metabolism, collateral pathways prove indispensable: the pentose phosphate pathway (PPP) and glycerol phosphate shuttle not only influence redox balance but sustain pro-inflammatory phenotypes by supplying substrates for epigenetic modifications [26]; while sterol regulatory element-binding protein 1 (SREBP1)-mediated de novo lipogenesis (DNL) impairs antioxidant defences by depleting NADPH, thereby promoting ROS-driven alternative macrophage activation [27]. Moreover, transient receptor potential (TRP) channels have been identified as “sensors” linking metabolic states to immune responses. By detecting lipids, redox signals, and metabolites such as lactate, they regulate calcium signalling, thereby remodelling mitochondrial function and glycolytic flux [28]. Even the intracellular complement system (complosome) has been demonstrated to be pivotal in regulating metabolic reprogramming, with its activity being crucial for inducing glycolysis and OXPHOS [29].

Based on the aforementioned microscopic mechanisms, immunometabolism proposes a core hypothesis: systemic metabolic disorders can reprogramme the metabolic pathways of immune cells, endowing them with a persistent pro-inflammatory phenotype. This process is often termed ‘trained immunity’ or innate immune memory, wherein immune cells undergo long-term epigenetic and metabolic reprogramming following initial exposure to endogenous metabolic stimuli (such as oxidised low-density lipoprotein, hyperglycaemia, uric acid, etc.), leading to excessive inflammatory responses to subsequent challenges [30,31]. In diseases such as atherosclerosis, sepsis, and heart failure, altered nutrient availability in the microenvironment (e.g., ischaemia, lipotoxicity) compels immune cells to undergo metabolic adaptation. This adaptation frequently induces mitochondrial dysfunction and ROS accumulation, thereby driving chronic inflammation and tissue damage [23,32,33]. For instance, metabolic stress following myocardial injury recruits immune cells and induces their polarisation towards a pro-inflammatory phenotype, establishing a non-resolving chronic inflammatory state [32,34]. Thus, metabolic reprogramming is not merely a by-product of immune activation but serves as a bridge linking systemic metabolic disorders (e.g., obesity, CVD) with immune dysregulation [33,35].

### 1.3. Trained Immunity and Metabolic Memory

While immunometabolic reprogramming describes the immediate cellular response, its long-term consequences manifest as distinct immunological and clinical states. Traditional immunological perspectives held that immune memory was an exclusive feature of the adaptive immune system. However, recent research has overturned this understanding, introducing the concept of ‘trained immunity’. This represents a form of non-specific immune memory formed by innate immune cells (such as monocytes, macrophages, neutrophils, and NK cells) following transient exogenous or endogenous stimulation [36,37,38,39]. This memory enables innate immune cells to mount enhanced inflammatory responses upon subsequent, even unrelated, secondary stimuli [40,41]. Unlike adaptive immunity, which relies on gene rearrangement, the mechanism underlying trained immunity is based on extensive metabolic reprogramming and epigenetic remodelling [39,42]. Specifically, transient stimuli (such as high glucose, oxidised low-density lipoprotein, or microbial products) trigger intracellular metabolic switches, leading to sustained activation of pathways including glycolysis, glutamine breakdown, and cholesterol synthesis [17,31,37]. These metabolic alterations not only furnish cells with energy and biosynthetic precursors but, crucially, enable metabolic intermediates (e.g., acetyl-CoA, lactate, fumarate) to participate as cofactors in histone modifications (e.g., acetylation, methylation, lactylation). This process ‘fixes’ the open state of pro-inflammatory genes at the chromatin level [31,43,44]. This ‘metabolic-epigenetic’ coupling mechanism enables cells to maintain a long-term ‘pre-activated’ or ‘hyper-responsive’ state even after the initial stimulus has dissipated [41,45].

In metabolic disorders, the training of immune mechanisms explains why cardiovascular complications persist even when blood glucose or lipid levels are controlled—a phenomenon termed “metabolic memory” [46,47,48]. Research indicates that transient hyperglycaemic stimulation suffices to induce a persistent pro-inflammatory phenotype in haematopoietic stem/progenitor cells (HSPCs) within the bone marrow and their differentiated macrophage counterparts [47,49]. This hyperglycaemia-induced training of immunity relies upon sustained glycolytic enhancement and activation of the transcription factor Runx1, leading to increased chromatin accessibility. This facilitates the release of greater pro-inflammatory factors when cells encounter subsequent stimuli [49]. Similarly, hyperlipidaemia—particularly oxidised low-density lipoproteins and cholesterol crystals—acts as a training inducer, triggering metabolic and epigenetic reprogramming in HSPCs. This promotes myeloid-biased bone marrow production and generates monocytes with heightened inflammatory potential [50,51]. These “trained” immune cells not only persist in the circulation but also migrate into atherosclerotic plaques, accelerating lesion progression and instability [17,49]. Notably, this metabolic memory is not confined to mature immune cells but, crucially, occurs at the level of HSPCs within the bone marrow—termed “central trained immunity” [52,53]. This implies that metabolic dysfunction leaves a profound imprint at the haematopoietic origin, causing successive generations of immune cells to carry this ‘pathogenic memory,’ thereby driving chronic low-grade inflammation and cardiovascular diseases [53,54].

Trained immunity is a double-edged sword. In anti-infection and tumour immunity, appropriate trained immunity (such as that induced by β-glucan or BCG) can enhance host defences and even overcome the immunosuppression of the tumour microenvironment [55,56]. However, in chronic inflammatory diseases such as diabetes and atherosclerosis, ‘maladaptive trained immunity’ induced by metabolic dysregulation acts as a driver of disease progression [50,52,54]. Consequently, the precise regulation of trained immunity becomes pivotal for therapeutic intervention. On one hand, interventions targeting the metabolic-epigenetic axis (such as inhibiting glycolysis or blocking specific histone-modifying enzymes) hold promise for erasing the epigenetic marks of trained immunity to disrupt pathological metabolic memory and alleviate chronic inflammation [46,49,57]. Conversely, inducing beneficial trained immunity through nanomaterials or specific metabolites (e.g., resorcinol) may offer novel strategies for infection control and tissue repair [58,59]. Future research must further elucidate the specific metabolic–epigenetic profiles triggered by different stimuli to develop targeted therapies capable of distinguishing between ‘beneficial’ and ‘harmful’ memories [36,41].

### 1.4. The Main Thrust of This Review Article

This paper aims to construct a multidimensional pathophysiological framework elucidating the underlying mechanisms of residual risk in CVD. Its core logical chain originates from glyco-lipid toxicity. Hyperglycaemia and dyslipidaemia are not merely metabolic substrate overload, but act as pivotal signalling molecules initiating pathological cascades within immune cells [47,49]. This metabolic stress transcends the conventional notion of mere ‘fuel’, becoming an initiator of immune activation by disrupting mitochondrial function and inducing oxidative stress [46,48]. Notably, the hyperglycaemic environment has been demonstrated to maintain a pro-inflammatory phenotype via trained immunity, establishing a long-lasting metabolic memory even after blood glucose returns to normal levels. This explains why cardiovascular risk remains elevated in diabetic patients despite stringent glycaemic control [46,49]. 

At the core of this pathological chain lies the tight coupling between intracellular metabolic reprogramming and epigenetic modifications. Upon sensing metabolic stress, immune cells (particularly monocytes/macrophages) undergo metabolic rewriting characterised by enhanced glycolysis, disrupted tricarboxylic acid cycle, and altered fatty acid synthesis [37,42]. These metabolic alterations lead to the accumulation of specific intermediates (e.g., lactate, acetyl-CoA), which serve as cofactors or substrates for epigenetic modifying enzymes. These enzymes directly modify histones (e.g., through histone acetylation), thereby reshaping chromatin accessibility [39,43,44]. This mechanism induces ‘trained immunity’—a long-lasting, non-specific innate immune memory—enabling immune cells to mount explosive inflammatory responses upon subsequent challenge [41].

This pathological process is not confined to the local vascular environment but undergoes systemic amplification through cross-organ communication networks. Recent evidence indicates that metabolic dysregulation and peripheral inflammatory signals can remotely regulate the bone marrow niche, inducing persistent transcriptional and epigenetic alterations in haematopoietic stem and progenitor cells (HSPCs)—termed “central training immunity” [51,60]. This bone marrow-level reprogramming induces myelopoietic bias, continuously releasing ‘trained’ monocytes and neutrophils with high inflammatory potential into the circulation [17,50,51]. These cells act as carriers, transporting the imprint of trained immunity from the bone marrow to cardiac and vascular tissues. This systemic immune amplification mechanism constitutes a pivotal bridge linking metabolic diseases (such as periodontitis, arthritis, and diabetes) to cardiovascular complications [50].

The ultimate consequence of the aforementioned cascade reaction is accelerated cardiovascular remodelling. Immune cells bearing metabolic memory, upon infiltrating vascular walls or damaged myocardium, drive atherosclerotic plaque instability and promote vascular calcification and myocardial fibrosis through sustained release of pro-inflammatory factors and matrix-degrading enzymes, ultimately precipitating major adverse cardiovascular events [17,36]. Building upon this logical chain, this review will delve into potential therapeutic strategies targeting the ‘metabolic–epigenetic’ axis. It proposes that, beyond conventional lipid-lowering and glucose-lowering therapies, targeting immune metabolic reprogramming and reversing immune training may represent novel breakthroughs for eliminating residual CVD risk and achieving precision cardiovascular protection [36,58].

## 2. Glucolipotoxicity: The Metabolic Trigger of Immune Impairment

### 2.1. Sugar Toxicity: More than Just Excess Energy

In environments of impaired glucose metabolism, immune cells undergo significant metabolic reprogramming, exhibiting a preference for aerobic glycolysis (the Warburg effect) as their primary energy source even under oxygen-sufficient conditions. This metabolic shift not only fulfils the ATP demands of rapid cellular proliferation but also supports biomolecular synthesis and regulates inflammatory phenotypes [61,62,63]. Research confirms that hyperglycaemic conditions induce enhanced glycolytic flux in macrophages and synovial macrophages, a process frequently accompanied by upregulation of glucose transporters (e.g., GLUT1) [49,64]. Hyperglycaemic glycolysis leads to accumulation of the intermediate metabolite lactate, which subsequently activates pathogenic genes at the epigenetic level through histone lactylation modifications (e.g., H4K12la, H4K8la), thereby driving retinal neovascularisation and osteoarthritis progression [65,66]. Moreover, this glycolysis-dependent metabolic state is closely linked to HIF-1α stability, forming a feedback loop that sustainably maintains pro-inflammatory cytokine secretion (e.g., IL-1β) and M1-type macrophage polarisation [67,68,69]. In atherosclerotic plaques, glycolytic enzyme expression levels correlate strongly with disease activity, further confirming the central role of the Warburg effect in driving chronic vascular inflammation [63].

When intracellular glucose is in excess, a portion of glucose is diverted to the hexosamine biosynthesis pathway (HBP), yielding UDP-GlcNAc, the substrate for O-GlcNAc glycosylation modification of proteins. O-GlcNAcyltransferase (OGT), acting as a key nutrient sensor, translates hyperglycaemic signals into post-translational modifications of intracellular proteins, thereby regulating cellular function [70]. In diabetic retinopathy models, retinal endothelial cells exhibit markedly elevated O-GlcNAc modification levels. This modification occurs directly at the threonine 383 site of the YAP protein, a key effector in this pathway, inhibiting its phosphorylation and degradation. Consequently, YAP becomes abnormally activated, inducing vascular dysfunction [71]. A similar mechanism underlies diabetic foot ulcer pathogenesis, where hyperglycaemia-induced abnormal O-GlcNAc modification impedes granulation tissue formation and epithelial re-epithelialisation, impairing wound healing [72]. Furthermore, in mouse models of type 2 diabetes combined with breast cancer, tumour tissues exhibit elevated O-GlcNAc modification levels, suggesting this pathway serves as a crucial molecular bridge linking metabolic dysregulation to pathological progression [73].

Prolonged hyperglycaemia promotes non-enzymatic reactions between reducing sugars and proteins or lipids, yielding advanced glycation end products (AGEs). Excessive accumulation of AGEs within the body constitutes a key pathogenic factor in diabetic complications such as diabetic nephropathy and retinopathy [74,75]. By binding to the specific receptor RAGE on cell surfaces, AGEs initiate a cascade of signalling reactions, activating transcription factors such as NF-κB and AP-1. This leads to explosive production of ROS and sustained release of inflammatory mediators [74,76,77]. Activation of this axis not only disrupts the glomerular filtration barrier and endothelial cell function but also induces a vicious cycle through mitochondrial dysfunction and oxidative stress [75,78]. In obesity and metabolic syndrome, RAGE/DIAPH1 axis interactions further exacerbate adipose tissue inflammation and insulin resistance. Blocking AGEs-RAGE interactions or inhibiting RAGE expression has been demonstrated to effectively mitigate inflammatory responses, improve diabetic wound healing, and mitigate renal injury, indicating that this axis represents a key downstream pathway for glycation toxicity-induced immune impairment [79,80].

### 2.2. Lipotoxicity: Lipids as Danger-Associated Molecular Patterns

Under metabolic disorder conditions, free saturated fatty acids (SFAs), particularly palmitate, act as potent endogenous danger-associated molecular patterns (DAMPs), directly triggering a sterile inflammatory cascade. Research confirms that palmitate exposure not only causes endothelial cell damage but also induces severe endothelial inflammation and adhesion responses. This process relies on activation of the MAP4K4/NF-κB signalling pathway, thereby promoting leukocyte homing and infiltration [81]. Beyond direct receptor activation, intracellular palmitate accumulation induces severe organelle stress. For instance, in macrophages, lipid overload triggers endoplasmic reticulum (ER) stress via the ATF4 branch, impairing exocytosis and lysosomal dysfunction, thereby obstructing inflammatory resolution [82]. Moreover, in cardiomyocytes, exposure to high palmitate concentrations induces explosive mitochondrial reactive oxygen species (mtROS) production and mitochondrial DNA (mtDNA) release, thereby activating the cGAS-STING signalling pathway. This further amplifies the inflammatory response through cardiomyocyte-macrophage communication [83].

As atherosclerotic plaques progress, free cholesterol precipitates to form cholesterol crystals (CCs). These crystals are no longer inert deposits but key drivers inducing plaque instability [84,85]. Acting as physical danger signals, CCs, when phagocytosed by macrophages, induce lysosomal rupture and mitochondrial dysfunction. This shifts ATP production towards anaerobic glycolysis and generates substantial ROS [86]. This metabolic and oxidative stress state directly activates the NLRP3 inflammasome, leading to the maturation and release of IL-1β and triggering pyroptosis. Pyroptosis is a form of programmed cell death characterised by membrane pore formation and the release of inflammatory mediators, further exacerbating the formation of necrotic cores within plaques [87,88]. Targeting this mechanism, nanotechnology-mediated dissolution of CCs within plaques has been demonstrated to effectively inhibit the TLR4-NF-κB pathway, reduce macrophage infiltration, and stabilise plaques [89].

**Figure 1:**
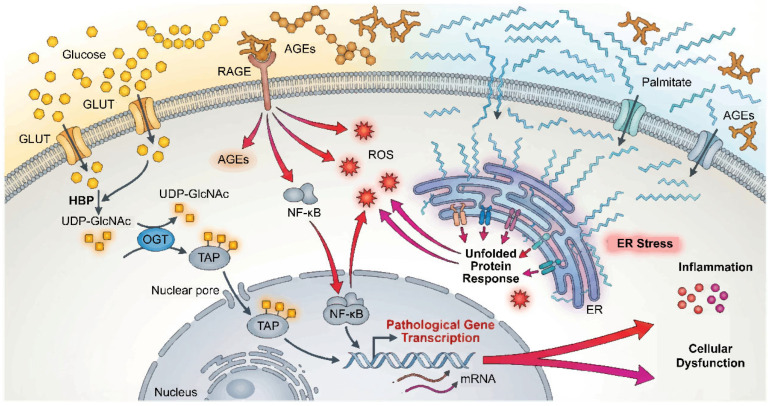
**Primary Metabolic Stress Triggered by Glucolipotoxicity.** High glucose levels promote the hexosamine biosynthetic pathway (HBP), increasing UDP-GlcNAc levels, which undergo nuclear translocation. Concurrently, advanced glycation end-products (AGEs) bind to their receptor (RAGE), triggering ROS production and NF-κB activation. In the endoplasmic reticulum (ER), lipid overload (palmitate) induces ER stress and the unfolded protein response (UPR). These converging pathways drive pathological gene transcription in the nucleus, ultimately leading to cellular dysfunction and chronic inflammation.

Oxidised low-density lipoprotein (oxLDL) and its carried OxPLs constitute persistent drivers of chronic inflammation within the vascular wall [15]. OxPLs initiate pro-atherosclerotic signalling pathways by interacting with scavenger receptors such as CD36 and LOX-1 [90,91]. Within endothelial cells, oxLDL induces specific matrix-dependent endoplasmic reticulum stress (e.g., fibronectin) and amplifies pro-inflammatory gene expression via the JNK-c-Jun signalling axis, leading to endothelial dysfunction [91,92]. Concurrently, oxLDL uptake disrupts macrophage phagocytic function, upregulates LOX-1 expression, and establishes a positive feedback loop that accelerates lipid phagocytosis and foam cell formation [93,94]. Moreover, oxLDL-mediated signalling pathways activate the transcription factor FOXS1, thereby suppressing the expression of the cholesterol efflux transporter (ABCA1/ABCG1). This leads to intracellular lipid accumulation and sustained activation of the NLRP3 inflammasome, ultimately driving vascular and valvular calcification processes [87], as shown in Figure 1.

### 2.3. Mitochondrial Dysfunction: The Powerhouse of Inflammation

Mitochondria, serving as the cellular ‘powerhouse,’ transform into ‘generators’ of inflammatory responses under pathological conditions characterised by metabolic substrate overload (e.g., high-fat diets, diabetes, ischaemia-reperfusion). When the electron transport chain (ETC) becomes overloaded due to metabolic stress, electron leakage triggers explosive production of mtROS [95,96,97]. Excessive mtROS not only directly causes oxidative damage but also acts as a second messenger, activating inflammasomes (such as NLRP3) to promote the maturation and release of pro-inflammatory factors (e.g., IL-1β) [95,96,98]. Moreover, mtROS further damages mitochondria through feedback mechanisms, leading to a loss of mitochondrial membrane potential, impaired respiratory function, and reduced ATP production, thereby establishing a vicious cycle [99,100,101]. In cardiomyocytes, this oxidative stress directly triggers apoptosis and necrosis, resulting in cardiac dysfunction and remodelling [100,101,102].

Another critical consequence of mitochondrial damage is the release of mitochondrial DNA (mtDNA). Crucially, as recent evidence emphasises, mtDNA is never released ‘alone’; rather, it is mobilised as part of a coordinated pro-inflammatory cocktail of mitochondrial damage-associated molecular patterns (mtDAMPs) facilitated by excessive mitochondrial fission and subsequent structural collapse. Under conditions such as oxidative stress or mitochondrial dyskinesis (e.g., Drp1-mediated excessive fission), the mitochondrial network undergoes extensive fragmentation. This process often culminates in increased mitochondrial outer membrane permeability (MOMP) or the assembly of large pores, which permit the synchronised co-release of mtDNA alongside other potent DAMPs, including cytochrome c, cardiolipin, and N-formyl peptides, from the mitochondrial matrix into the cytoplasm or extracellular environment [103,104,105]. While mitochondrial ROS generation is a well-established driver of cardiovascular inflammation, the specific role of cytoplasmic mtDNA acting as a ‘danger signal’ recognised by cyclic GMP-AMP synthase (cGAS) represents an emerging paradigm. This pathway activates the stimulator of interferon genes (STING) signalling cascade, inducing robust type I interferon responses and pro-inflammatory cytokine expression, a mechanism currently supported primarily by compelling preclinical evidence [103,104,106]. Concurrently, the co-released molecules collectively amplify the activation of other innate immune sensors—such as the NLRP3 inflammasome by cardiolipin and ROS—creating a multi-component ‘inflammatory eruption’ rather than a simple, isolated leak of genetic material. This mechanism plays a central role in multiple cardiovascular and metabolic diseases: in heart failure and myocardial infarction, mtDNA released from cardiomyocytes can be phagocytosed by macrophages, promoting their M1-type polarisation and exacerbating inflammation and tissue damage [83,103,104]; in atherosclerosis, abnormal mtDNA synthesis in plaque macrophages also intensifies inflammation via the STING pathway; in senescent cells, mtDNA leakage is a key driver of the senescence-associated secretory phenotype (SASP) [105]. Interventions targeting the mtDNA-cGAS-STING axis (such as STING inhibition or promoting autophagy-mediated mtDNA clearance) have demonstrated significant therapeutic potential across multiple animal models, emerging as promising new targets for blocking sterile inflammation [83,107].

**Figure 2:**
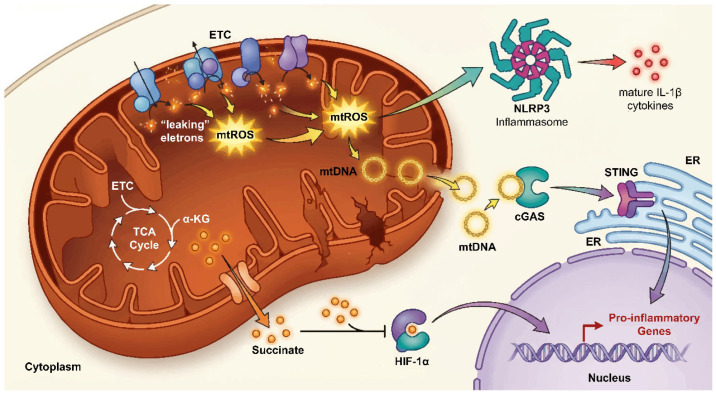
**Mitochondria: From “Energy Hub” to “Inflammatory Dynamo”.** In dysfunctional mitochondria, the TCA cycle is disrupted, leading to the accumulation of succinate, which stabilises HIF-1α to promote pro-inflammatory gene expression. “Leaking” electrons generate mitochondrial ROS (mtROS), a potent activator of the NLRP3 inflammasome, resulting in IL-1β maturation. Additionally, the release of mitochondrial DNA (mtDNA) into the cytoplasm triggers the cGAS-STING pathway, further amplifying the sterile inflammatory response.

Mitochondrial metabolic dysfunction and inflammatory flare-ups are frequently accompanied by the failure of mitochondrial quality control mechanisms. Under normal conditions, cells selectively eliminate damaged mitochondria through mitophagy to maintain the health of the mitochondrial network [106,108,109]. However, under pathological conditions (such as ischaemia-reperfusion injury, high-fat diet, or ageing), the autophagic flux becomes impaired, leading to the accumulation of damaged mitochondria and mtDNA [106,110]. For instance, the absence of PINK1-mediated autophagy exacerbates stress-induced cardiac hypertrophy and inflammation [106], while TFAM not only participates in mtDNA maintenance but also acts as an autophagy receptor to promote cytoplasmic mtDNA clearance (i.e., ‘nucleolar autophagy’), thereby limiting inflammatory responses [109]. Restoring mitochondrial quality control, such as through nanomedicines that scavenge ROS, enhance autophagy, or modulate mitochondrial dynamics, has been demonstrated to effectively mitigate myocardial injury and inflammation [101,102,107], as shown in Figure 2.

### 2.4. Insulin Resistance: Failure of the Immune Brake

Under physiological conditions, insulin not only regulates blood glucose homeostasis but also serves as a crucial immune ‘braking’ mechanism. Upon binding to its receptor (Insulin Receptor, IR), insulin activates the PI3K-Akt signalling pathway, thereby promoting glucose uptake and metabolism while effectively suppressing the activity of pro-inflammatory transcription factors such as FOXO1 and NF-κB [111,112,113]. This signalling mechanism is crucial for maintaining the quiescent state of vascular smooth muscle cells (VSMCs), endothelial cells, and immune cells [112,113,114]. For instance, in VSMCs, normal insulin signalling inhibits Thrombospondin-1 (Thbs1) and Matrix metalloproteinase-2 (Mmp2) expression via the Akt-FoxO1 axis, thereby reducing inflammatory cytokine secretion, suppressing apoptosis, and maintaining plaque stability [112].

However, in metabolic disorders such as obesity and type 2 diabetes, systemic or tissue-specific insulin resistance renders this anti-inflammatory mechanism ineffective. When insulin signalling is impaired, reduced activity of the PI3K-Akt pathway releases inhibition on transcription factors such as FOXO1. Hyperactivated FOXO1 translocates to the nucleus, initiating the transcription of pro-inflammatory genes and resulting in the overexpression of cytokines (e.g., TNF-α, IL-6) and adhesion molecules (e.g., VCAM-1, ICAM-1) [112,114]. Insulin resistance characteristics and increased expression of FOXO signalling-related genes have been observed in the nuclei of cardiomyocytes from congenital heart disease (CHD) patients, suggesting the prevalence of this mechanism in cardiac pathophysiology [113]. Furthermore, the PD-1 receptor on macrophages has been found to be closely linked to metabolic function. Under conditions of immune checkpoint blockade therapy or high-fat diet induction, blocking or downregulating PD-1 signalling exacerbates metabolic disorders, whereas activated PD-1 signalling exerts anti-inflammatory effects by inhibiting IRE1α-mediated endoplasmic reticulum stress [115]. This indicates that insulin resistance represents not merely a failure of glucose regulation, but also the loss of a crucial endogenous defence mechanism within the immune system. This loss perpetuates chronic low-grade inflammation and accelerates the progression of cardiovascular complications [112,116,117].

## 3. Metabolic Remodelling of Immune Cell Subpopulations

### 3.1. Innate Immunity: The Plasticity and Phagocytosis of Macrophages

The immune function of macrophages is tightly coupled with their intracellular metabolic state. This reshaping of ‘immunometabolism’ determines their phenotypic polarisation and functional fate within the atherosclerotic environment. Macrophages rely on specific metabolic reprogramming to execute their functions; for instance, pro-inflammatory M1 macrophages typically exhibit enhanced glycolysis, whereas anti-inflammatory, reparative M2 macrophages depend more heavily on FAO and mitochondrial oxidative phosphorylation (OXPHOS) [18]. Research indicates that lipid metabolism is not merely a consequence of macrophage polarisation but also a driving factor [118]. For instance, ALDH2 deficiency accelerates cGAS degradation by inhibiting its interaction with USP14, thereby obstructing cGAS-STING pathway activation. This leads to pro-inflammatory macrophage polarisation and diminished anti-inflammatory capacity [119]. Conversely, epigenetic modifications such as MeCP2 methylation at the K271 site, which interacts with H3K36me3 to inhibit RUNX1 transcription, promote M2-type macrophage polarisation and enhance plaque stability [120]. Furthermore, the transcription factor KLF14 has been demonstrated to exert anti-atherosclerotic effects by promoting cholesterol efflux through upregulating ABCA1 while simultaneously suppressing inflammatory cascades [121]. Probiotic-derived extracellular vesicles (PEVs) have also been found to induce macrophage phenotype conversion from M1 to M2 by regulating pathways including glycolysis, OXPHOS, and amino acid metabolism [18].

In the mechanism of foam cell formation, lipid uptake receptors form a vicious cycle with intracellular oxidative stress signalling. Abnormal expression of scavenger receptors LOX-1 and CD36 is pivotal in lipid overload. The ubiquitin ligase TRIM31 has been identified as a protective factor that promotes K48-linked ubiquitination and degradation of LOX-1, thereby limiting foam cell formation [122], whereas another ubiquitin ligase, RNF128, promotes oxLDL-induced foaming by stabilising the SRB1 receptor [123]. Mitochondrial metabolism plays a central role in this process. OxLDL-induced mitochondrial superoxide drives STAT5 activation, which in turn inhibits TCA cycle activity, forming a ‘ROS-STAT5’ positive feedback loop that accelerates macrophage differentiation into foam cell [124]. Moreover, Ca^2+^ influx mediated by the mechanosensitive channel Piezo1 triggers mitochondrial ROS production and activation of the NF-κB/MAPK pathway, whereas the natural antioxidant kaempferol mitigates foam cell formation by blocking this axis [125].

**Figure 3:**
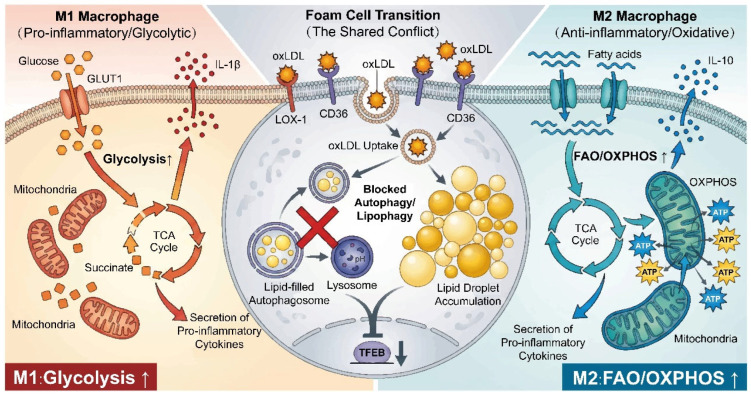
**The “Immuno-metabolic Switch” and Macrophage Foam Cell Formation.** M1 macrophages utilise aerobic glycolysis to support pro-inflammatory cytokine secretion, while M2 macrophages rely on fatty acid oxidation (FAO) and oxidative phosphorylation (OXPHOS) for anti-inflammatory functions. In the context of atherosclerosis (Foam Cell Transition), the excessive uptake of oxLDL via CD36/LOX-1, coupled with blocked autophagy/lipophagy and TFEB inhibition, leads to lipid droplet accumulation and the formation of foam cells within the vascular wall.

The ultimate formation of foam cells is frequently attributed to impaired lipid degradation pathways—particularly autophagy and lipophagy. Dysfunction of lysosomes, serving as metabolic sensors and signalling platforms for immune metabolic reprogramming, constitutes one hallmark of atherosclerosis [126]. Studies reveal that the nuclear receptor Dax1, highly expressed in plaque macrophages, inhibits autophagy by directly interacting with TFEB (a key autophagy regulator), leading to lipid accumulation [127]. Similarly, the acid-sensitive ion channel ASIC1 interacts with RIP1, promoting phosphorylation of both RIP1 and TFEB, thereby disrupting TFEB-mediated lipophagy [128]. To counteract this pathological process, enhancing autophagic flux emerges as a potential therapeutic strategy: overexpression of ATG14 promotes autophagosome–lysosome fusion, reversing oxLDL-induced autophagic blockage [129]; Fucoidan [130] and the vitamin D3-VDR-PTPN6 axis [131] have both been demonstrated to restore lipophagy function and promote cholesterol efflux by upregulating TFEB or related autophagy genes. Recent proteomics studies have also systematically identified multiple lipid droplet-associated proteins, including SQSTM1/p62 and NBR1, as participants in macrophage lipophagocytosis, offering novel molecular targets for foam cell intervention [132], as shown in Figure 3.

### 3.2. Innate Immunity: Neutrophils and Immunothrombosis

Within the pathological context of diabetes and metabolic disorders, neutrophils serve not only as the primary responders in inflammatory reactions but also as pivotal mediators linking metabolic abnormalities to thrombotic risk. Hyperglycaemia has been demonstrated to be a potent driver of neutrophil extracellular trap formation (NETosis). Research indicates that hyperglycaemia induces excessive ROS production within neutrophils. This oxidative stress state triggers the release of intracellular calcium-binding proteins (such as S100A8/A9), thereby initiating NET formation [133]. Beyond classical biochemical pathways, the mechanosensitive ion channel Piezo1 exhibits upregulation in neutrophils from type 2 diabetic patients. Hyperglycaemia activates Piezo1 transcription, inducing discrete prothrombotic cellular responses and revealing a novel mechanothrombotic pathway regulated by the metabolic environment [134]. Furthermore, proteomic analyses indicate that high-glucose stimulation induces specific post-translational modifications (PTMs) in neutrophils. These modifications are closely associated with NET formation and metabolic alterations, further substantiating the remodelling effect of high-glucose environments on neutrophil function [135,136].

NETs comprise not only depolymerised chromatin DNA and histones (such as citrullinated histone H3, CitH3), but are also rich in granular proteins, including neutrophil elastase (NE) and myeloperoxidase (MPO). These structures act as physical scaffolds within blood vessels, trapping platelets and coagulation factors to promote the formation of ‘immune thrombi’ [137,138]. In the diabetic milieu, neutrophil–platelet interactions are recognised as a core mechanism driving thromboinflammation [139]. NET release not only amplifies the coagulation cascade but also alters fibrin clot architecture, inducing hypofibrinolysis in type 2 diabetic patients and impeding thrombus degradation [140,141]. Notably, circulating cathepsin G, a neutrophil-derived prothrombotic protease, has been demonstrated as an independent predictor of cardiovascular events in type 2 diabetes patients, further reinforcing the link between neutrophil activation and atherothrombosis [142].

**Figure 4:**
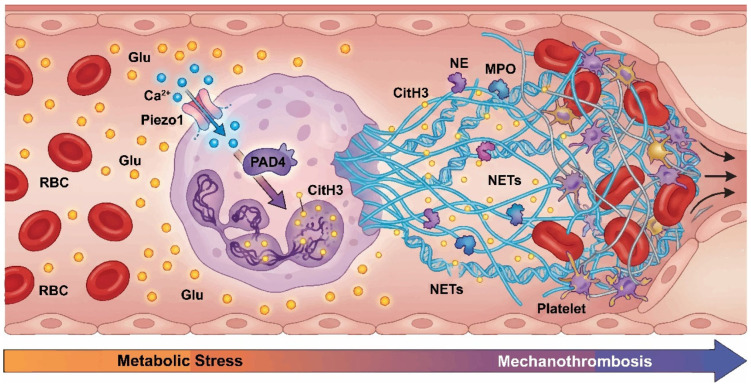
**Neutrophil NETosis and Immunothrombosis in Diabetes.** In a high-glucose environment, neutrophils sense mechanical or metabolic stress through the Piezo1 channel. This triggers Ca^2+^ influx and activates PAD4, leading to histone citrullination (CitH3) and the release of Neutrophil Extracellular Traps (NETs). These NETs act as a scaffold, trapping red blood cells (RBCs) and platelets, thereby promoting mechanothrombosis and vessel occlusion.

High-glucose-induced NETosis carries severe pathological consequences in cardiovascular complications such as diabetic cardiomyopathy (DCM) and acute coronary syndrome (ACS). Experimental evidence indicates that diabetes drives inflammation and fibrosis in the heart and kidneys by promoting neutrophil inflammasome activation and NETosis; knockout of poly(L-lysine)-dependent deimidases 4 (PAD4, a key NET-forming enzyme) significantly alleviates diabetes-induced heart failure and protects renal function, suggesting that NETs are a key factor in diabetic cardio-renal injury [143]. In an obesity-induced hyperglycaemic stroke model, interaction between neutrophil surface integrin α9 and cellular fibronectin promotes NETosis via the ERK/PAD4 pathway, exacerbating thromboinflammatory responses and cerebral infarct volume following reperfusion [144]. Although most studies support high glucose promoting NET formation, others highlight disease heterogeneity. For instance, circulating NET markers did not significantly increase in long-term type 1 diabetes patients, suggesting functional plasticity of neutrophils across different disease courses and metabolic contexts [145]. Nevertheless, overall, targeting NET formation inhibition or promoting their degradation has emerged as a significant potential strategy for alleviating diabetes-associated immune thrombosis and microvascular complications [146,147], as shown in Figure 4.

### 3.3. Adaptive Immunity: Metabolic Checkpoints of T Cells

The intensity and nature of adaptive immune responses are subject to stringent regulation by intrinsic metabolic programmes within T cells. Within the pathological microenvironment of cardiovascular disease, systemic metabolic dysregulation (such as hyperlipidaemia and hyperglycaemia) interacts with local metabolic stress to reshape T-cell metabolic checkpoints. This leads to the expansion of pathogenic T-cell subsets and the depletion of protective subsets.

The differentiation of CD4+ T cells towards pro-inflammatory Th17 or anti-inflammatory Treg cells is highly dependent on the metabolic trade-off between glycolysis and OXPHOS/FAO. Research confirms that excessive activation of the glycolytic pathway is a key metabolic hallmark driving Th17 cell pathogenicity. In autoimmune myocarditis models, phosphoglycerate kinase 1 (PGK1)—a pivotal glycolytic enzyme—exhibits markedly elevated expression. Inhibiting PGK1 not only suppresses glycolytic activity but also blocks Th17 cell differentiation and alleviates myocardial fibrosis by modulating the pyruvate dehydrogenase kinase 1 (PDHK1)-mitochondrial reactive oxygen species (ROS) axis [148]. Furthermore, the transcription factor Bmi-1 limits pathogenic γδT17 cell generation and delays cardiac ageing by ubiquitinating and degrading RORγt, thereby inhibiting its nuclear transcriptional activity [149]. Silicate biomaterials have also been found to suppress excessive CD4+ T cell differentiation towards Th17 cells by modulating the FOXO signalling pathway, thereby interrupting the vicious cycle of myocardial inflammation [150].

In contrast, the stability of Treg cells relies more heavily on the homeostasis of lipid metabolism. In patients with severe coronary artery disease (CAD), APOB-reactive CD4+ T cells exhibit a loss of Treg transcriptional characteristics, instead acquiring glycolytic and interferon-responsive features. This metabolic reprogramming correlates positively with disease severity [151]. Further clinical and animal studies indicate that dyslipidaemia induced by hepatic injury is closely associated with reduced FOXO3 expression and increased IL-17A production in APOB-specific T cells, suggesting that systemic lipid metabolism disorders directly disrupt peripheral immune tolerance [152]. However, propionate—a gut microbiota metabolite—acts as an immunometabolic modulator. By increasing Treg numbers and IL-10 levels in the intestinal microenvironment while suppressing cholesterol transporter Npc1l1 expression, it improves lipid profiles and reduces atherosclerotic lesions [153]. Moreover, CD73+ Treg cells play a pivotal role in post-myocardial infarction repair, with alterations in their purinergic signalling pathways directly influencing cardiac inflammation resolution and remodelling [154]. Notably, in arrhythmogenic cardiomyopathy (ACM), Treg cells exhibit increased numbers yet display abnormal IL-32 overexpression, promoting lipid droplet accumulation in mesenchymal stromal cells. This suggests that Tregs may undergo metabolic and functional ‘defection’ within specific pathological contexts [155].

The role of CD8+ T cells in atherosclerosis and myocardial injury is dual-regulated by their intracellular lipid homeostasis and mitochondrial function. Alterations in the lipid environment represent not merely passive cellular stress but active regulatory signals [156]. Intracellular cholesterol accumulation is a key factor inducing T cell dysfunction; T cell-specific deletion of the cholesterol efflux transporter ABCA1/ABCG1 leads to intracellular cholesterol overload, subsequently inducing T cell apoptosis and accelerating senescence. Whilst this partially reduces T cell numbers within plaques, it also compromises immune surveillance functions [157].

Mitochondrial dysfunction serves as an intrinsic cellular trigger for the transition of CD8+ T cells from “precursor exhaustion” to “terminal exhaustion”. Oxidative stress induced by mitochondrial dysfunction inhibits the proteasomal degradation of HIF-1α, thereby promoting glycolytic reprogramming and driving T cells into a state of terminal exhaustion [158]. Within atherosclerotic plaques, PD-1+ T cells predominantly exhibit a precursor exhaustion phenotype (PD-1int) while retaining IFN-γ production capacity, primarily sustaining function via glycolysis. Such cells may be reactivated under immune checkpoint inhibitor therapy, potentially increasing cardiovascular risk [159]. In models of type 2 diabetes complicated by myocardial infarction, metabolic dysregulation leads to increased infiltration of CD8+ T cells in the infarct zone. These cells exhibit impaired mitochondrial function and upregulated chemokine expression, exacerbating adverse cardiac remodelling [160]. Moreover, doxorubicin-induced cardiotoxicity has been demonstrated to be CD8+ T cell-mediated, involving T cell infiltration via cardiac fibroblast ICAM-1 upregulation and granzyme B-dependent cardiomyocyte apoptosis [161]. In acute myocarditis, CD57+, CD8+ effector T cells—possessing high cytotoxicity and migratory potential—amplify via IL-18 signalling, emerging as a key subgroup driving myocardial injury [162].

### 3.4. Adaptive Immunity: B Cells and Autoantibodies

B cells play a dual role within the immune microenvironment of atherosclerosis, their function being largely determined by the heterogeneity of B cell subsets and the types of antibodies they secrete. B-1 cells and marginal zone B cells (MZB) primarily generate innate IgM antibodies targeting oxidation-specific epitopes (OSEs), which exert anti-atherosclerotic effects by neutralising oxLDL [163,164,165]. However, with disease progression or within specific pro-inflammatory microenvironments, the adaptive immune response may undergo pathogenic conversion. Follicular B cells enter the germinal centre reaction, undergo class switching, and produce high-affinity IgG antibodies against novel antigens such as MDA-LD [166,167]. This humoral immune activation is influenced not only by systemic metabolic states but may also be driven by the local microenvironment. Advanced atherosclerotic plaques may harbour arterial tertiary lymphoid organs (ATLOs) within their adventitia, structures that serve as neuro-immune-cardiovascular interfaces and locally generate disease-associated autoantibodies [168].

The core hazard of pathological autoantibodies lies in the formation and deposition of immune complexes (ICs). Research indicates that β2-glycoprotein I (β2GPI), as a multifunctional plasma protein, can bind with oxLDL and neutrophil extracellular traps (NETs) to form complexes. These complexes expose hidden epitopes, triggering the production of anti-β2GPI autoantibodies. The resulting IgG-type immune complexes deposit within vascular walls, activating and damaging endothelial cells, disrupting lipid metabolic homeostasis, and further undermining immune tolerance, thereby accelerating plaque progression [169]. Moreover, following myocardial infarction (MI), alarmins released from necrotic myocardium activate B cells via TLR-dependent pathways, promoting their terminal differentiation into plasma cells and extensive IgG secretion. These IgGs accumulate within plaques, accelerating atherosclerotic progression through Fc receptor-mediated inflammatory amplification mechanisms or complement system activation [170].

Although autoantibodies against certain modified autoantigens (such as methylglyoxal-modified ApoB100) demonstrate potential cardiovascular protective effects [171], overall, immune complex deposition mediated by anti-oxLDL autoantibodies constitutes a key driver of chronic vascular wall inflammation. This mechanism extends beyond lipid antigens to encompass immune responses against endogenous proteins. For instance, while autoantibodies against ALDH4A1 exhibit some degree of protective effect, it has been demonstrated that multiple antigens within plaques can trigger extensive B-cell clonal expansion [167]. Consequently, B-cell-mediated humoral immunity constitutes a crucial immunological basis for atherosclerotic plaque instability through antibody-dependent mechanisms, particularly by forming immune complexes deposited in the vascular wall and activating the complement cascade, as shown in Table 1.

## 4. Key Molecular Mechanisms: Sensing and Execution

### 4.1. NLRP3 Inflammasome: Integrator of Metabolic Danger

The NLRP3 inflammasome, as the core sensor of the innate immune system, is activated strictly following the “double-signaling model” to ensure an accurate response to metabolic danger signals. The first signal (initiating signal) is mainly mediated by the NF-κB pathway and is responsible for upregulating the transcriptional expression of NLRP3 and Pro-IL-1β. Various metabolic stressors can trigger this process, including lipid oxidation overload [172], exposure to environmental particulates [173], and the S100A8/A9 alarmin through the recognition-like protein (JAML) [174,175], which enhances NF-κB activity by promoting the nuclear translocation of PKM2 [176]; long non-coding RNA MIR181A1HG amplifies the NF-κB signal in endothelial cells by isolating the transcriptional repressor Foxp1 [177]. Additionally, the accumulation of NADH driven by glycolysis can lead to the oligomerisation of CtBP1, relieving its inhibitory effect on NLRP3 transcription and establishing a direct link between the cellular metabolic state and the initiation of inflammation [178].

The second signal (activation signal) pertains to disturbances in intracellular homeostasis, directly triggering the assembly of the NLRP3-ASC-Caspase-1 complex. This process integrates multiple subcellular stress signals:

1. Mitochondrial dysfunction and mtDNA release: The generation of mtROS serves as a key driver [179]. Specifically, METTL4-mediated abnormalities in N6-methyladenosine (6 mA) modification of mtDNA impair the activity of mitochondrial complex V and result in mtDNA release into the cytoplasm, directly activating inflammasomes [180,181].
2. Interactions between metabolic enzymes and ion channels: The dissociation of hexokinase 2 from VDAC on the mitochondrial outer membrane, leading to VDAC oligomerisation and calcium influx, has been demonstrated as a common upstream step in NLRP3 assembly [182].
3. Membrane receptors and lipid sensing: Specific metabolites such as the lipid peroxidation product octanal activate intracellular signalling cascades promoting inflammasome assembly via the olfactory receptor Olfr2/OR6A2 [183], while long-chain ceramides do so through GPCR receptors (CYSLTR2 and P2RY6).
4. Mechanical force transduction: Pathological shear forces may stabilise METTL14 via the mechanosensitive circular RNA (sno-circCNOT1), thereby activating NLRP33 [184], whereas pulsed electromagnetic fields reduce membrane tension by modulating TRPV4 channels, thereby inhibiting inflammasome activation [185].
5. The assembly and activity of the NLRP3 complex are subject to stringent post-transcriptional and epigenetic modifications. HDAC9-mediated deacetylation [186] and BRCC3-mediated deubiquitination [187] are prerequisites for NLRP3 activation. Conversely, chaperone-mediated autophagy (CMA) maintains low NLRP3 expression by recognising and degrading the NLRP3 protein via LAMP-2A; CMA dysfunction constitutes a key mechanism for NLRP3 overactivation in atherosclerosis [188]. Mutations in the epigenetic enzyme TET2 (commonly observed in clonal haematopoiesis) promote NLRP3 deubiquitination by enhancing JNK1 activity [187,189], whereas the metabolite α-ketoglutarate (AKG) suppresses inflammasome activity in vascular calcification by upregulating TET2 [190].
6. Upon activation, the NLRP3 inflammasome activates Caspase-1, which executes two core effects: firstly, cleaving Pro-IL-1β and Pro-IL-18 into mature bioactive forms, triggering an aseptic inflammatory storm [191]; secondly, cleaving Gasdermin D (GSDMD), inducing cell membrane perforation and pyroptosis [192]. The release of cellular contents due to pyroptosis not only exacerbates the formation of necrotic cores within plaques [193,194], but also promotes neutrophil recruitment and the formation of neutrophil extracellular traps (NETs) through the release of IL-1β. This establishes a vicious ‘inflammasome-NETosis’ cycle, significantly driving the progression of cardiovascular metabolic diseases [195].

### 4.2. Epigenetic Reprogramming: The Vehicle of Metabolic Memory

Metabolic signals serve not only as substrates for energy and biosynthesis, but also as pivotal factors profoundly reshaping the epigenetic landscape of immune cells. This “metabolic-epigenetic” interplay not only governs the immediate function of cells, but also permanently entrenches disease phenotypes by inducing trained immunity, culminating in systemic metabolic memory.

Intracellular metabolic flux directly regulates chromatin accessibility. Acetyl-CoA, serving as a pivotal hub in glycolipid metabolism, acts as a substrate for histone acetyltransferases (HATs). In macrophages and T cells, elevated acetyl-CoA levels generated via glycolysis and fatty acid oxidation drive high histone acetylation (e.g., H3K27ac), maintaining open states at pro-inflammatory gene promoters [175,196]. For instance, in LKB1-deficient cells, activation of the CRTC2-CREB signalling pathway recruits p300/CBP, elevating H3K27ac levels at inflammatory gene loci to sustain high pro-inflammatory potential under metabolic stress [197]. While histone acetylation and DNA methylation are well-established epigenetic regulators with extensive clinical validation, histone lactylation represents a newly emerging, metabolism-dependent modification. Predominantly demonstrated in recent preclinical models, it has been shown to be directly regulated by lactate levels, a glycolytic product. Following myocardial infarction, monocytes uptake lactate via MCT1 and upregulate histone lactylation (e.g., H3K18la), thereby activating the expression of reparative genes such as VEGF-A and IL-10 to promote cardiac repair [198]. Conversely, in vascular smooth muscle cells, H4K16 lactylation promotes PDK1 transcription and lactate accumulation via a positive feedback loop, exacerbating aortic aneurysm formation [199]. This demonstrates that metabolite-driven epigenetic modifications exhibit dual effects that are both cell-type and context-specific.

The remodelling of DNA methylation patterns constitutes another crucial vehicle for metabolic memory. The TET enzyme family (TET1/2/3) governs DNA demethylation, with its activity being highly dependent on α-ketoglutarate (α-KG), an intermediate metabolite of the tricarboxylic acid cycle. Clonal haematopoiesis induced by TET2 mutations (CHIP) constitutes an independent risk factor for cardiovascular disease. This mechanism arises from TET2 loss-of-function causing hypermethylation of DNA, which in turn renders macrophages hypersensitive to inflammatory stimuli [200,201]. Metabolic states directly influence TET enzyme function: for instance, α-KG supplementation suppresses vascular calcification by upregulating TET2 expression and enhancing its activity, thereby inhibiting NLRP3 inflammasome activation [190]. Conversely, in non-alcoholic fatty liver disease, DNMT1-mediated hypermethylation suppresses PPARα and HNF4α transcription, exacerbating lipid metabolism disorders [202]. Furthermore, environmental factors such as smoking induce hypomethylation at the F2RL3 locus, subsequently upregulating PAR4 expression and increasing platelet reactivity. This epigenetic alteration establishes a long-term memory of cardiovascular risk [203]. In Treg cells, defects in the non-oxidative pentose phosphate pathway lead to reduced α-KG levels, triggering DNA hypermethylation that compromises their immunosuppressive function and induces autoimmune diseases [204].

**Figure 5:**
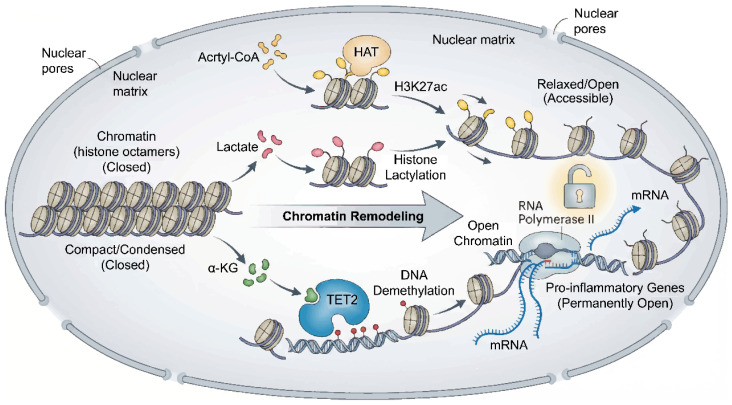
**Epigenetic Map: Metabolite-Mediated “Trained Immunity” and Metabolic Memory.** This figure illustrates how intracellular metabolites dictate gene accessibility. Acetyl-CoA facilitates histone acetylation (H3K27ac) via HATs; lactate promotes histone lactylation; and α-KG serves as a cofactor for TET2-mediated DNA demethylation. These modifications shift chromatin from a compact/closed state to an open/accessible state, allowing RNA polymerase II to sustain the transcription of pro-inflammatory genes.

As shown in Table 2, metabolic signals also influence immune cell differentiation and memory formation via chromatin remodelling complexes. The progressive integration of IL-12 and antigen signals remodels the chromatin landscape through STAT4 and AP-1 transcription factors, determining the differentiation fate of CD8+ T cells towards effector or memory phenotypes [205]. Within atherosclerotic plaques, histone acetyltransferases p300 and CBP synergistically regulate smooth muscle cell phenotypic conversion. Here, p300 interacts with TET2 to promote a contractile phenotype, whereas CBP recruits HDACs to inhibit this process [206]. This metabolic-epigenetic coupling mechanism explains why transient metabolic disturbances (such as hyperglycaemia or high-fat diets) leave a long-lasting ‘memory’ in immune cells, persisting as cardiovascular risk factors even after metabolic indicators return to normal, as shown in Figure 5.

### 4.3. Hypoxia-Inducible Factor (HIF-1α): The Metabolic–Inflammatory Hub

In the pathological progression of cardiovascular and metabolic diseases, hypoxia-inducible factor-1α (HIF-1α) has transcended its traditional role as a mere oxygen sensor, establishing itself as a central hub in the ‘metabolic–inflammatory’ axis. Not only does it respond to hypoxia, but it is also stably expressed under non-hypoxic metabolic stress signals (such as hyperglycaemia, succinate accumulation, and oxidised lipid overload), thereby coordinating cellular metabolic reprogramming and inflammatory storms.

HIF-1α is a key transcriptional regulator of glycolytic conversion (the Warburg effect) in immune cells. In macrophages and T cells, HIF-1α directly upregulates the expression of crucial glycolytic genes, including glucose transporter 1 (GLUT1), hexokinase 2 (HK2), and fructose-1,6-bisphosphate-2-kinase (PFKFB3) [207,208,209]. This metabolic reorganisation is crucial for maintaining the pro-inflammatory phenotype. For instance, TREM-1 activation promotes HIF-1α nuclear translocation via the PI3K/AKT/mTOR pathway, thereby enhancing glycolysis and suppressing oxidative phosphorylation—a process essential for NLRP3 inflammasome activation [210]. Moreover, phosphorylation of the glycolytic rate-limiting enzyme PFKL has been demonstrated to elevate HIF-1α protein levels, establishing a positive feedback loop linking glycolysis, HIF-1α, and inflammation [211]. In models of bioprosthetic valve calcification and myocardial injury, both the Acod1-Hif-1α-glycolysis axis within macrophages and AIF1-mediated HIF-1α activation have been shown to exacerbate tissue inflammation and adverse remodelling by driving glycolytic metabolic flux [212,213]. HIF-1α directly translates intracellular metabolic accumulation into inflammatory output, with its regulation of IL-1β representing the most pivotal mechanism. Under pathological conditions (such as ischaemia or inflammation), the metabolic intermediate succinate accumulates abnormally, inhibiting prolyl hydroxylases (PHDs) to block HIF-1α degradation. Stable HIF-1α not only promotes inflammatory gene transcription but also directly binds to the IL-1β promoter region, driving precursor production [214,215]. Research has established the decisive role of the ‘succinate/HIF-1α/IL-1β’ signalling axis in accelerating atherosclerotic plaque progression [215]. Furthermore, lysosomal dysfunction (such as LAMP2A deficiency) leads to elevated HIF-1α protein levels, thereby promoting VEGF-A and IL-1β secretion and inducing angiogenesis and inflammatory responses [216].

**Table 2:** Key metabolites/cofactors and their corresponding epigenetic modifications and pathogenic phenotypes.

Metabolite	Enzyme/Site	Epigenetic Effect	Impact on Immune Genes	CV Pathology	PMID
Lactate	H3K18la (histone lactylation)	Promoting the transcription of repair genes	Upregulation of VEGF-A, IL-10	Promoting myocardial repair following infarction (M2 polarisation)	[198]
	H4K16la/p300	Enhancing promoter accessibility	Promotes PDK1 transcription (positive feedback)	Metabolic remodelling of vascular smooth muscle cells exacerbates aortic aneurysms	[199]
Acetyl-CoA	H3K27ac/p300/CBP	Open chromatin	Maintain high expression of pro-inflammatory genes	Macrophages remain persistently activated, maintaining metabolic memory.	[175,197]
α-Keto-pentanoic acid (α-KG)	TET2 (DNA demethylase)	Maintain low DNA methylation	Inhibition of NLRP3 inflammasome activation	Alleviate vascular calcification; reverse the risk of clonal haematopoiesis	[190,201]
Succinate	PhDs (inhibition)/HIF-1α	Stabilise HIF-1α protein	Directly binds to and drives IL-1β transcription	Accelerating inflammation in atherosclerotic plaques	[214,215]

Within the complex microenvironment of atherosclerotic plaques, HIF-1α also acts as an integrator of lipid metabolism disorders and immune activation. Oxidised low-density lipoprotein (oxLDL) and reactive oxygen species (ROS) can induce HIF-1α stabilisation, which in turn promotes triglyceride and cholesterol synthesis within macrophages while inhibiting cholesterol efflux, forming a vicious cycle [217]. Targeted inhibition of HIF-1α restores the distribution of cholesterol transporters (ABCA1/ABCG1), promotes cholesterol efflux, and reduces foam cell formation [218]. More significantly, HIF-1α serves as a pivotal node linking metabolic stress to NLRP3 inflammasome activation; under conditions of homocysteinemia or hyperglycaemic environments, inhibiting HIF-1α significantly blocks TLR4/NF-κB/NLRP3 pathway activation, thereby mitigating pyroptosis and inflammatory storms [210].

## 5. Systemic Inter-Organ Crosstalk: A Network of Systemic Communication

### 5.1. Heart–Adipose Axis: The Adipokine Duet

The homeostasis of the cardiovascular system is not only regulated by the local microenvironment but is also deeply embedded within the metabolic networks of systemic adipose tissue. The cardio-adipose axis establishes a complex cross-organ interaction network through paracrine and endocrine signalling, wherein the functional state of adipose tissue—whether “beneficial” or “deleterious”—directly determines the pathological outcomes of the heart and vasculature.

Epicardial adipose tissue (EAT) shares a microcirculation with the myocardium without anatomical barriers, constituting the heart’s most immediate metabolic and immunological microenvironment. Under pathological conditions (such as obesity, diabetes, or heart failure), EAT undergoes profound remodelling, transforming from a physiologically protective layer into a pro-inflammatory, pro-fibrotic pathogenic source [219,220,221]. EAT serves as a primary source of pro-inflammatory cytokines. In patients with heart failure with preserved ejection fraction (HFpEF) and atrial fibrillation (AF), EAT exhibits increased volume and reduced density (decreased CT values), closely associated with heightened macrophage and neutrophil infiltration [220,222]. Factors secreted by EAT, such as IL-1β, IL-6, and TNF-α, diffuse directly into the adjacent myocardium, inducing cardiomyocyte hypertrophy, apoptosis, and microvascular rarefaction, leading to diastolic dysfunction [223,224]. Furthermore, immune cell activation within EAT (such as the enrichment of CD8+ tissue-resident memory T cells) can directly modulate calcium influx in cardiomyocytes, increasing susceptibility to arrhythmias [225]. Pro-fibrotic factors secreted by EAT (e.g., TGF-β1, MMP9) and adipokines (e.g., Leptin, Resistin) promote atrial fibrosis and structural remodelling, providing a matrix for the onset and maintenance of atrial fibrillation [222,223]. Leptin further enhances myocardial CaMKII activity by activating the sympathetic release of neuropeptide Y (NPY), thereby triggering arrhythmias. Conversely, the protective factor PRG4 shows reduced secretion in EAT of atrial fibrillation patients [223]. Alterations in EAT volume and density constitute independent predictors of HFpEF and coronary artery disease progression [219,226,227]. For instance, reduced EAT density (indicating increased inflammation) correlates significantly with the progression of non-calcified plaque burden [228]. Novel therapeutics such as SGLT2 inhibitors and GLP-1 receptor agonists have been demonstrated to reduce EAT volume and ameliorate its inflammatory state, thereby exerting cardiovascular protective effects [229,230].

Perivascular adipose tissue (PVAT) envelops most blood vessels except those of the brain, forming the “fourth layer” of the vascular wall. Its physiological functions include secreting “adipose-derived relaxing factors” (ADRFs, such as adiponectin and omentin-1) to counteract vasoconstriction and inflammation. However, in obesity and metabolic disorders, PVAT undergoes functional dysregulation and phenotypic transformation [231,232].

In obesity and type 2 diabetes, the anti-contractile effects of perivascular adipose tissue (PVAT) are diminished. This is attributed to reduced secretion of protective adipokines (such as adiponectin and omentin-1) alongside increased secretion of pro-contractile and pro-inflammatory factors (such as chemerin and IL-6) [233,234]. This imbalance promotes proliferation, migration, and phenotypic switching of vascular smooth muscle cells (VSMCs), leading to increased vascular stiffness and hypertension [234,235]. PVAT exhibits browning characteristics, a trait crucial for maintaining vascular homeostasis. Vascular injury induces browning in PVAT, which secretes factors such as Neuregulin 4 (Nrg4) to promote macrophage polarisation towards the anti-inflammatory M2 phenotype, thereby accelerating inflammation resolution and vascular repair [236]. Conversely, Prdm16 deficiency induces PVAT ‘white fat’ transformation and loss of browning, exacerbating vascular fibrosis and elevated blood pressure via QSOX1-mediated mechanisms [235]. PVAT serves as a reservoir for diverse immune cells, including macrophages, T cells, and B-1 cells. Under obesity conditions, myeloperoxidase (MPO)-positive myeloid cells accumulate within PVAT, inhibiting brown adipogenesis and impairing endothelial function [237]. Conversely, the Pdpn+ macrophage subpopulation secretes lipid mediators via the Pla2g2d-GPR120 axis, exerting vasoprotective effects and improving insulin resistance [238].

### 5.2. Heart–Liver Axis: Hepatogenic Inflammation

The liver and heart maintain close bidirectional communication under both physiological and pathological conditions, forming the “cardio-hepatic axis”. Metabolism-associated steatohepatitis (MASLD, formerly termed non-alcoholic fatty liver disease) represents not merely a localised hepatic metabolic disorder but also an independent risk factor for cardiovascular disease (CVD). Its core mechanism lies in the liver’s role as a metabolic hub: when compromised, it releases multiple hepatokines and inflammatory mediators that trigger distal cardiac injury.

During the progression of MASLD to metabolic-associated steatohepatitis (MASH), the liver becomes the “engine” of systemic inflammation. Damaged hepatocytes and activated Kupffer cells release substantial pro-inflammatory factors, including C-reactive protein (CRP), interleukin-6 (IL-6), tumour necrosis factor-α (TNF-α), and fetuin-A [239,240,241].

**Figure 6:**
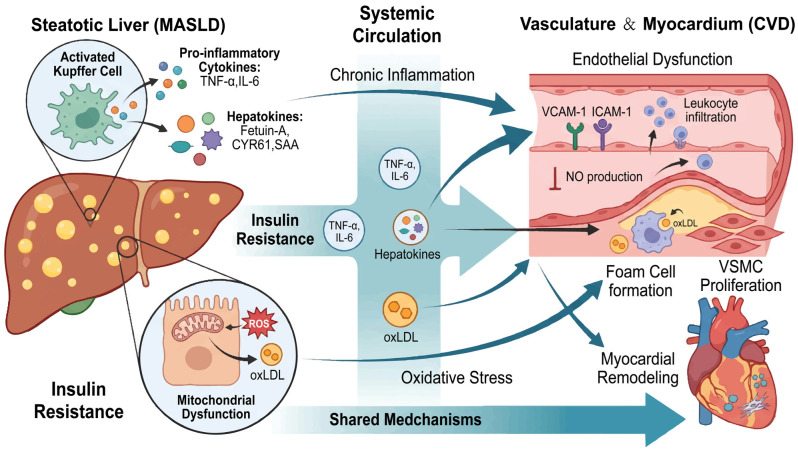
**The Cardio-Hepatic Axis: Shared Pathological Mechanisms and Inter-organ Crosstalk.** The progression of metabolic-associated steatotic liver disease (MASLD) acts as an “engine” for systemic inflammation and cardiovascular disease (CVD). The relationship is characterised by shared core mechanisms: (1) Insulin Resistance reduces endothelial NO production; (2) Oxidative Stress drives the generation of circulating oxLDL; (3) Chronic Inflammation, fuelled by activated Kupffer cells, releases TNF-α and IL-6; and (4) Endothelial Dysfunction facilitates leukocyte infiltration. Concurrently, liver-derived hepatokines (e.g., Fetuin-A, CYR61, and SAA) act on the myocardium and vasculature via endocrine signalling to induce insulin resistance, myocardial remodelling, and atherogenesis.

MASLD and CVD are intimately connected not only through cross-organ communication but also because they share multiple core pathological mechanisms, fundamentally driven by systemic metabolic dysregulation (Figure 6). Specifically, insulin resistance, a hallmark of MASLD, disrupts hepatic lipid homeostasis while simultaneously impairing insulin signalling pathways (e.g., PI3K/Akt) in the vascular endothelium. This impairment reduces endothelial nitric oxide (NO) bioavailability and triggers compensatory hyperinsulinemia, which synergistically promotes vascular smooth muscle cell (VSMC) proliferation and plaque vulnerability [239]. Concurrently, hepatic lipid overload induces mitochondrial dysfunction and severe oxidative stress. The excessive production of reactive oxygen species (ROS) from the steatotic liver exacerbates systemic lipid peroxidation, generating circulating oxidised low-density lipoproteins (oxLDL) that act as primary drivers for macrophage foam cell formation in the arterial wall [242].

Furthermore, the steatotic liver serves as a central hub for chronic low-grade inflammation. Sustained activation of hepatic Kupffer cells leads to the continuous release of pro-inflammatory cytokines (such as TNF-α, IL-6, and CRP) into the systemic circulation. These inflammatory mediators directly induce endothelial dysfunction by upregulating the expression of adhesion molecules (e.g., VCAM-1, ICAM-1), thereby facilitating circulating leukocyte tethering, rolling, and subendothelial infiltration [239,242]. Within this complex cross-talk, specific hepatokines and metabolic axes act as molecular bridges exacerbating CVD. For instance, liver-derived Fetuin-A links steato-toxicity to insulin resistance by acting as an endogenous ligand for Toll-like receptor 4 (TLR4), activating the NF-κB pathway in cardiac tissues. This induces local inflammation and insulin resistance, thereby exacerbating cardiac metabolic dysfunction [240,241]. Similarly, serum amyloid A (SAA) may contribute to the pathogenesis of HFpEF by promoting endothelial dysfunction and myocardial fibrosis [243]. Hepatic steatosis is also associated with cardiovascular risk via the leukopoietic–arterial axis, where metabolically impaired livers stimulate the pro-inflammatory differentiation of hematopoietic stem cells in the bone marrow, increasing the number of inflammatory leukocytes in the circulation [244]. Moreover, the liver-derived CYR61 protein has been demonstrated to polarise macrophages towards a pro-fibrotic/pro-inflammatory phenotype, which not only exacerbates hepatic fibrosis but may also influence cardiac remodelling via the circulation [245]. Furthermore, EVs serve as novel communication carriers, transporting bioactive molecules (such as miRNAs and proteins) to mediate metabolic and immune signals between the liver and cardiovascular system, thereby regulating systemic inflammation and vascular health [246]. Interventions targeting these shared mechanisms, such as SGLT2 inhibitors and GLP-1 receptor agonists, have demonstrated potential for concurrently improving hepatic and cardiac outcomes [247].

### 5.3. Cardio-Hematopoietic Axis: Central Regulation

The bone marrow is the wellspring of cardiovascular health. With advancing age, haematopoietic stem cells (HSCs) in the bone marrow progressively accumulate somatic mutations, leading to the development of clonal haematopoietic ageing (CHIP). CHIP has emerged as a key independent risk factor for cardiovascular disease (CVD), with the most prevalent mutated genes including DNMT3A and TET2 [248]. Myeloid cells differentiated from HSCs harbouring these mutations exhibit abnormal pro-inflammatory properties. For instance, TET2-deficient macrophages, due to aberrant epigenetic modifications, demonstrate extreme sensitivity to inflammatory signals such as IL-1β. This leads to NLRP3 inflammasome overactivation, thereby accelerating the progression and instability of atherosclerotic plaques [17]. Moreover, CHIP is not only associated with coronary artery disease but also closely linked to poor heart failure prognosis, increased atrial fibrillation risk, and heightened recurrence and mortality following ischaemic stroke [201,249]. Clinical data indicate that the presence of CHIP can even predict cardiovascular mortality risk in asymptomatic carotid stenosis patients [250], and correlates with poorer short-term outcomes in cardiogenic shock and acute myocardial infarction patients [251]. Notably, factors such as premature menopause, obesity, and chronic inflammation may also promote CHIP development by influencing HSC clonal selection [252,253].

**Figure 7:**
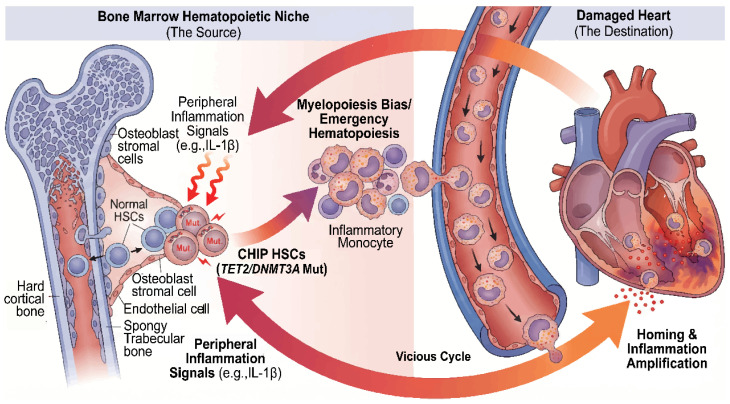
**Systemic Communication Map: Bone Marrow Hematopoietic Niche and Central Trained Immunity.** Heart injury releases peripheral inflammatory signals (e.g., IL-1β) that act on the bone marrow niche. In individuals with CHIP (clonal haematopoiesis of indeterminate potential), mutations in TET2 or DNMT3A lead to a myelopoiesis bias, generating an excess of inflammatory monocytes. These cells home to the damaged heart, amplifying local inflammation and further exacerbating cardiovascular remodelling.

Acute myocardial infarction (MI) is not merely a local cardiac event but also triggers a systemic “emergency bone marrow haematopoietic” response, creating a vicious cycle. Post-MI sympathetic activation and systemic inflammatory signals (such as IL-1β and IL-6) stimulate bone marrow HSCs to proliferate, leading to excessive production of monocytes and neutrophils [254]. These newly generated inflammatory cells subsequently home to the injured heart. While initially aiding in the clearance of necrotic tissue, excessive inflammatory infiltration exacerbates myocardial injury and adverse remodelling, potentially accelerating atherosclerotic progression and causing a ‘secondary hit’ [254,255]. Research indicates that platelet-derived extracellular vesicles (pEVs) released post-MI, particularly those carrying miR-499, can be taken up by HSCs, promoting their proliferation and myeloid differentiation, thereby further intensifying the inflammatory response [255]. Moreover, the spleen—as a site of extramedullary haematopoiesis—becomes activated post-MI, not only generating substantial inflammatory cells but also participating in monocyte storage and release [256]. Inhibiting this pathological emergency haematopoiesis, for instance by targeting retinoic acid metabolism or suppressing IL-1 signalling pathways, has been demonstrated to effectively mitigate post-MI inflammation and enhance cardiac repair [256]. Interventions targeting the cardio-myelo axis offer novel therapeutic avenues for CVD management. Anti-inflammatory agents such as colchicine have demonstrated potential in clinical trials to mitigate cardiovascular risks associated with TET2-mutant CHIP and inhibit clonal expansion [257]. Furthermore, targeted therapies addressing specific mutation-downstream inflammatory pathways (e.g., IL-6, NLRP3) in DNMT3A mutations, alongside nanotechnology-based specific modulation of the bone marrow microenvironment or macrophage function [258], present promising applications. Understanding and intervening in this axis holds significant importance for disrupting the inflammatory drivers of CVD, as shown in Figure 7.

### 5.4. Heart–Gut Axis: Microbial Metabolites

The gut microbiota, functioning as an “invisible organ”, engages in extensive molecular dialogue with the host cardiovascular system through its metabolic products. Disruption of this communication—known as dysbiosis—not only compromises intestinal barrier function but also leads to abnormal translocation of bioactive molecules, directly modulating vascular inflammation, blood pressure homeostasis, and myocardial remodelling.

Leaky gut represents the initial phase of pathophysiological interaction within the gut–brain axis. Impairment of the intestinal barrier facilitates translocation of lipopolysaccharide (LPS), a component of Gram-negative bacterial cell walls, into the bloodstream, thereby inducing metabolic endotoxaemia. As a potent virulence factor, LPS drives systemic low-grade inflammation by directly activating innate immunity or indirectly inducing non-microbial danger-associated molecular patterns (DAMPs), thereby accelerating atherosclerosis, obesity, and insulin resistance [259,260]. Regarding metabolic products, trimethylamine N-oxide (TMAO) is a recognised cardiovascular risk factor. High-fat diets disrupt mitochondrial bioenergetics in the colonic epithelium, increasing oxygen and nitrate utilisation within the intestinal lumen. This promotes the respiratory-dependent degradation of choline by Escherichia coli to produce TMA, which is ultimately converted to TMAO in the liver [261]. Elevated TMAO levels are closely associated with the development of pulmonary arterial hypertension, atrial fibrillation, and heart failure [262,263]. Beyond well-established metabolites like TMAO, recent studies have identified emerging microbial metabolites such as imidazole propionate (ImP), which is significantly elevated in patients with atherosclerosis. Based primarily on preclinical investigations, ImP directly drives inflammation and lesion progression by activating the imidazoline-1 receptor (I1R/Nischarin) on myeloid cells, without altering lipid levels [264,265]. Notably, while TMAO is harmful, its precursor TMA exhibits the capacity to inhibit IRAK4 kinase activity, thereby partially blocking TLR4 signalling pathways, suggesting the complexity of microbial metabolite actions [266].

In contrast to harmful metabolites, short-chain fatty acids (SCFAs) such as propionate and butyrate, produced by dietary fibre fermentation, act as protective factors for cardiovascular health. Propionate reduces cholesterol absorption and inhibits plaque progression by increasing regulatory T cells (Treg) and IL-10 levels in the intestinal microenvironment, thereby downregulating the expression of the intestinal cholesterol transporter Npc1l1 [153]. SCFAs act as ligands for the host G protein-coupled receptors GPR41 and GPR43. Deficiency in these receptors leads to elevated blood pressure, exacerbated cardiac and renal fibrosis, and increased intestinal permeability; supplementation with dietary fibre or SCFAs restores blood pressure homeostasis [267,268]. Butyrate has been demonstrated to inhibit NET formation, thereby mitigating pathological alterations in abdominal aortic aneurysms (AAA) [269]. Furthermore, in a Kawasaki disease vasculitis model, butyrate-producing bacteria or their metabolites significantly alleviated cardiovascular inflammation by enhancing the intestinal barrier [270]. In pre-eclampsia, reduced SCFAs correlate with macrophage autophagy defects; supplementation with propionate or butyrate promotes M2 polarisation and improves trophoblast cell invasion [271].

Beyond SCFAs, gut microbiota profoundly influence cardiac health through their metabolism of tryptophan and purines. Increased intestinal IDO1 enzyme activity redirects tryptophan metabolism away from protective indole derivatives towards harmful kynurenine and serotonin, the latter exacerbating intestinal barrier damage and atherosclerosis [272]. Conversely, the microbial metabolite indole-3-propionic acid (IPA) protects the heart from heart failure with preserved ejection fraction (HFpEF) and immune checkpoint inhibitor-induced cardiotoxicity by activating the aryl hydrocarbon receptor (AhR), thereby upregulating SIRT3 expression and improving mitochondrial function [273]. Inosine produced by the probiotic Bifidobacterium infantis exerts significant cardioprotective effects against myocardial ischaemia/reperfusion injury by activating the adenosine A2A receptor (A2AR) and inhibiting inflammatory cytokine production [274].

### 5.5. Heart–Muscle Axis: The Protection and Loss of Myokines

Skeletal muscle is not only a motor organ but also one of the body’s largest endocrine organs. It engages in extensive cross-organ dialogue with the heart by secreting a series of bioactive molecules known as myokines. In healthy states, exercise-induced myokines exert potent cardiovascular protective effects. Conversely, in ageing, obesity, or metabolic disorders, the loss of muscle mass and function—such as sarcopenic obesity—leads to a deficiency in protective myokines, thereby exacerbating cardiovascular risk.

Irisin, a cleavage product of the exercise-induced myofactor FNDC5, is hailed as the ‘exercise hormone’. It demonstrates remarkable cardioprotective efficacy in myocardial ischaemia/reperfusion injury, hypertensive cardiac remodelling, and diabetic cardiomyopathy. Irisin suppresses ROS production and NLRP3 inflammasome activation within macrophages, promoting M2 polarisation to mitigate myocardial inflammation and microvascular injury [275,276]. In diabetic cardiomyopathy models, irisin inhibits ferroptosis via the SIRT1-p53-SLC7A11/GPX4 pathway, protecting cardiomyocytes from hyperglycaemic toxicity [277]. Irisin improves endoplasmic reticulum stress and mitochondrial homeostasis by activating the AMPK pathway, thereby preventing cardiomyocyte apoptosis and fibrosis [278]. Exercise-induced irisin-rich extracellular vesicles (EVs) increase SIRT6 stability in vascular smooth muscle cells, delaying vascular stiffness and endothelial dysfunction [279]. Irisin also traverses the blood–brain barrier, promoting astrocytic release of neprilysin to degrade β-amyloid, thereby ameliorating Alzheimer’s disease-associated cognitive decline and demonstrating its role in ‘muscle-brain’ communication [280].

In addition to irisin, other myokines participate in the cardioprotective network: for instance, musclin counteracts stress-induced heart failure by enhancing C-type natriuretic peptide (CNP) signalling, promoting myocardial contraction and inhibiting fibroblast activation [281]. Resistance exercise stimulates skeletal muscle secretion of FSTL1, which promotes post-ischaemic myocardial vascularisation via the DIP2A-Smad2/3 pathway, thereby improving cardiac function [282]. The C-terminal peptide segment of CCDC80, secreted in response to exercise, specifically inhibits the JAK2/STAT3 pathway, mitigating hypertensive-induced cardiac hypertrophy and fibrosis [283].

Sarcopenic obesity denotes a pathological state where obesity coexists with sarcopenia, commonly observed in elderly patients with heart failure. This ‘double whammy’ not only signifies heightened lipotoxicity and chronic inflammation but also represents the depletion of skeletal muscle as a metabolic buffer and source of myokines. Adipose infiltration (intermuscular adipose tissue, IMAT) and the release of inflammatory mediators (e.g., IL-6) induced by obesity suppress muscle protein synthesis and mitochondrial function, accelerating muscle atrophy [284,285]. Conversely, diminished muscle mass leads to inadequate secretion of protective myokines (e.g., irisin, FGF4), impairing their regulatory capacity over systemic metabolism and the cardiovascular system [286]. Patients with sarcopenic obesity exhibit a markedly elevated risk of poor outcomes in heart failure with reduced ejection fraction (HFrEF), serving as an independent predictor of cardiovascular mortality [287]. Resistance training combined with high-intensity interval training (HIIT) has been demonstrated to improve mitochondrial function and redox balance in the elderly [288]. Furthermore, interventions targeting specific metabolic checkpoints (such as BCAA catabolism and sphingosine synthesis) offer novel therapeutic targets for enhancing muscle mass and cardiovascular health [289,290], as shown in Table 3.

### 5.6. Disease-Specific Dominance and Integrative Hierarchy of Organ Crosstalk

While these communication axes form a synergistic network driving cardiovascular immune injury, their relative importance and mechanistic dominance vary across different pathological contexts.

In atherosclerosis, the cardio-hematopoietic axis acts as the central amplifier and “memory carrier.” Clonal haematopoiesis and central trained immunity in the bone marrow provide the sustained supply of hyper-inflammatory myeloid cells that dictate plaque progression and instability [17,248]. This process is systemically fuelled by the heart–gut axis, where microbial metabolites like TMAO and leaky gut-derived LPS act as primary metabolic triggers [259,260,261].

In contrast, for heart failure (HF)—particularly HFpEF—and arrhythmias, the heart–adipose axis exerts a more immediate and dominant paracrine influence. Epicardial adipose tissue (EAT) functions as a local “inflammatory powerhouse,” where its proximity allows for direct cytokine infiltration (e.g., IL-1β, IL-6) into the myocardium, driving fibrosis and structural remodelling [220,222,223,224]. Concurrently, the heart–muscle axis serves as a systemic metabolic buffer; the loss of exercise-induced myokines like irisin in sarcopenic states often represents a tipping point for cardiac decompensation [276,286,287].

Therefore, these axes form a hierarchical structure: the gut and liver provide the systemic inflammatory “prime,” the bone marrow establishes the long-term inflammatory “memory,” and the adipose tissue facilitates the local “execution” of injury. Understanding this disease-specific relevance is essential for moving toward precision immunometabolic therapy.

## 6. Clinical Translation: Dual-Targeted Therapeutic Strategies

### 6.1. Immunomodulatory Effects of Metabolic Drugs

The mechanism of action of traditional metabolic drugs in cardiovascular protection has expanded beyond mere glucose and lipid reduction to encompass profound immunometabolic remodelling. Through unique molecular pathways, these medications regulate systemic metabolism while directly intervening in immune cell function, thereby exerting dual anti-inflammatory and anti-remodelling effects.

SGLT2 inhibitors (such as empagliflozin and dapagliflozin), serving as cornerstones in the treatment of heart failure and chronic kidney disease, derive their cardiovascular benefits largely from immunomodulatory effects. By elevating β-hydroxybutyrate (β-OHB) levels, these agents inhibit the assembly and activation of the NLRP3 inflammasome, thereby reducing the release of pro-inflammatory cytokines, including IL-1β and IL-6. This effect has been demonstrated in both diabetic and non-diabetic models of myocardial infarction, atherosclerosis, and renal injury [291,292,293]. By upregulating the SIRT1/AMPK signalling pathway, SGLT2 inhibitors enhance cellular autophagy flux, alleviate endoplasmic reticulum stress and oxidative stress, thereby inhibiting macrophage foaming and myocardial fibrosis [293,294]. SGLT2 inhibitors also directly inhibit the late sodium current (late-INa) in cardiomyocytes, improving sodium-calcium homeostasis and thereby reducing inflammation and arrhythmia risk [295].

GLP-1 receptor agonists (such as liraglutide and semaglutide) exhibit broad anti-inflammatory properties beyond glucose control and weight reduction. By inhibiting macrophage foaming, GLP-1RAs reduce lipid deposition and T-cell infiltration within vascular walls, thereby stabilising plaques and delaying lesion progression [296]. GLP-1 receptor agonist therapy also reduces reactive oxygen species (ROS) production, restores mitochondrial function, and diminishes leukocyte adhesion and rolling to endothelial cells. It lowers the expression of adhesion molecules such as ICAM-1 and VCAM-1, thereby improving vascular endothelial function [296,297]. In diabetic nephropathy, GLP-1 receptor agonists mitigate renal inflammation and injury by downregulating RAGE receptor expression, reducing myeloid progenitor cell proliferation in bone marrow, and promoting macrophage M2 polarisation [298].

Beyond activating AMPK, metformin also inhibits mitochondrial complex I, thereby reducing ROS production and subsequently suppressing the differentiation of pathogenic Th17 cells to exert anti-inflammatory effects [299]. Furthermore, it regulates energy balance by inducing the production of the anorexigenic metabolite N-lactoyl-phenylalanine (Lac-Phe) [300].

Statins not only reduce LDL-C but also exhibit significant anti-inflammatory pleiotropy. By inhibiting the isoprenylation process in the mevalonate pathway, they block the membrane localisation and activity of small G proteins (such as Rho/Rac), thereby suppressing inflammatory signalling pathways, including NF-κB [301]. Statins also enhance macrophage efferocytosis by suppressing CD47 expression, thereby promoting the clearance of necrotic cells within plaques [302]. Notably, although statins may increase diabetes risk, their cardiovascular benefits—including anti-inflammatory effects—remain uncompromised [303].

In summary, SGLT2 inhibitors, GLP-1 receptor agonists, metformin and statins provide a dual metabolic-immune therapeutic approach for cardiovascular metabolic diseases through an interwoven immunometabolic regulatory network involving multiple mechanisms.

### 6.2. Targeted Immunotherapy

Given the pivotal role of residual inflammation in cardiovascular disease, targeted interventions directed at specific immune pathways or mediators have emerged as a key strategy for reducing cardiovascular events.

Canakinumab is a monoclonal antibody targeting the pro-inflammatory cytokine IL-1β. The landmark CANTOS trial demonstrated that in patients with prior myocardial infarction and elevated hsCRP, Canakinumab significantly reduced the risk of major adverse cardiovascular events (MACE), independent of changes in lipid levels [304,305]. Notably, cardiovascular benefits were more pronounced in patients with chronic haematopoietic impairment (CHIP), particularly those with TET2 mutations, alongside improvements in anaemia. This suggests potential for precision medicine within specific high-risk genetic contexts [306,307]. However, this therapy did not reduce all-cause mortality and increased the risk of fatal infections, limiting its widespread clinical application [304].

IL-6 is a key inflammatory mediator downstream of IL-1β. Tocilizumab, an IL-6 receptor antagonist, demonstrated in patients with acute ST-segment elevation myocardial infarction (STEMI) that a single dose increased myocardial salvage fraction, reduced microvascular occlusion, and significantly diminished systemic inflammatory response (C-reactive protein and white blood cell count), with no serious adverse events observed [308,309]. Furthermore, tocilizumab effectively suppresses inflammation in patients with non-ST-segment elevation myocardial infarction (NSTEMI). The novel IL-6 receptor antagonist Ziltivekimab demonstrated significant reductions in hsCRP and other inflammatory and thrombotic markers in the Phase II RESCUE trial among patients with chronic kidney disease and high cardiovascular risk, with favourable safety profiles, thereby establishing a foundation for subsequent large-scale cardiovascular outcome trials [310].

Colchicine, as an ancient anti-inflammatory agent, exerts its effects through multiple mechanisms, including inhibition of microtubule polymerisation, interference with neutrophil chemotaxis, and suppression of NLRP3 inflammasome activation [311,312]. Several large-scale randomised controlled trials (e.g., COLCOT, LoDoCo2) and their meta-analyses confirm that low-dose colchicine (0.5 mg/day) significantly reduces the risk of major adverse cardiovascular events (MACE) in coronary heart disease patients (including those with chronic coronary syndrome and post-acute myocardial infarction), particularly myocardial infarction and stroke. This benefit is independent of statin therapy [313,314,315]. The FDA has approved its use for reducing cardiovascular event risk in patients with atherosclerotic cardiovascular disease [316]. Colchicine demonstrates greater cardiovascular benefit in myocardial infarction patients with concomitant type 2 diabetes mellitus [317]. Furthermore, for carriers of the TET2 mutation (CHIP), colchicine may provide additional protective effects by inhibiting clonal expansion and inflammation [318,319]. Despite efficacy in preventing ischaemic stroke and MACE, colchicine failed to meet primary endpoints in perioperative myocardial injury prevention [320] and recurrence prevention following acute ischaemic stroke (CONVINCE trial), frequently accompanied by gastrointestinal adverse reactions [320].

Beyond the aforementioned drugs, other strategies targeting inflammatory pathways are under investigation. For instance, nanotherapeutics targeting CD47 may resolve plaque inflammation by restoring efferocytosis in macrophages [321]; folate-modified bionic nanovesicles loaded with PU.1 inhibitors can target macrophages within plaques to suppress inflammation [99]; immunosuppressive therapy for myocarditis-like episodes has demonstrated potential for improving outcomes in patients with pathogenic desmin variants [322]. Furthermore, incorporating CHIP into risk assessment models may provide more precise anti-inflammatory treatment guidance for high-risk populations [323].

### 6.3. Immune Metabolic Reprogramming Medication

Current cardiovascular treatment strategies are progressively shifting from singular metabolic regulation or anti-inflammatory approaches towards a dual-pronged approach aiming to reverse immunometabolic reprogramming and eradicate the disease’s metabolic memory. A comprehensive summary of these strategies, explicitly categorised by their current translational stages (from experimental models to clinically approved therapies), is provided in Table 4.

In pathological processes such as atherosclerosis and myocardial fibrosis, metabolic reprogramming of macrophages and fibroblasts is characterised by enhanced glycolysis. Pyruvate kinase M2 (PKM2), as a key enzyme in glycolysis, plays a central role in driving inflammation and fibrosis through its non-metabolic functions (such as nuclear translocation). Selective PKM2 inhibitors (e.g., Compound 3K) and active components of traditional Chinese herbal medicines (e.g., ginseng-and-acorus injection, baicalin) not only block glycolytic flux by inhibiting the PKM2-HIF-1α axis but also reduce proinflammatory cytokine secretion and extracellular matrix deposition, thereby improving myocardial fibrosis and cardiac function [324,325]. Furthermore, inhibition of other glycolytic enzymes such as PFKFB3 has been demonstrated to suppress NLRP3 inflammasome activation in endothelial cells by reducing NADH accumulation and CtBP1 oligomerisation [207].

Restoring mitochondrial health represents another pivotal strategy for reversing immunometabolic dysregulation. Mdivi-1 (a mitochondrial fission inhibitor) not only suppresses pathological mitochondrial fission in vascular smooth muscle cells and macrophages but also promotes anti-inflammatory M2-type transformation by reprogramming macrophage metabolism, thereby reducing oxidative stress and foam cell formation [326,327]. Natural products such as naringin and the flavonoid wogonin enhance macrophage cholesterol efflux capacity and induce anti-inflammatory polarisation by activating the PPARα pathway, thereby promoting fatty acid oxidation (FAO) and inhibiting glycolysis [328,329]. SGLT2 inhibitors have also been found to exert broad cardiorenal protective effects by improving mitochondrial function through upregulating SIRT1 and AMPK, while inhibiting NLRP3 inflammasome activation [294].

**Figure 8:**
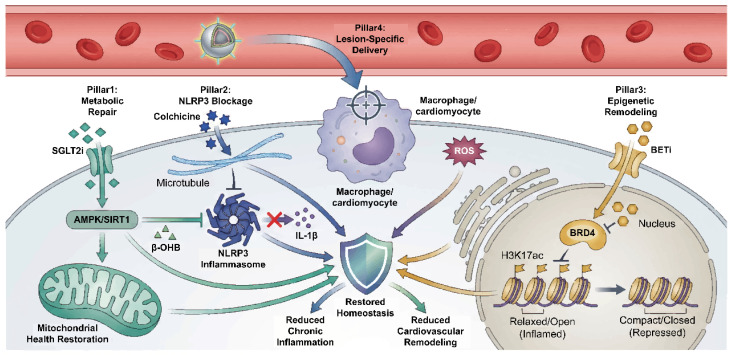
**Precision Intervention Path Targeting Immunometabolic Reprogramming**. Four therapeutic pillars are proposed: (1) Metabolic Repair via SGLT2i and AMPK/SIRT1 activation to restore mitochondrial health; (2) NLRP3 Blockage using agents like colchicine; (3) Epigenetic Remodelling with BET inhibitors to close pro-inflammatory chromatin; and (4) Lesion-Specific Delivery using targeted nanoparticles to improve drug efficacy in macrophages and cardiomyocytes.

Epigenetic imprints of metabolic dysregulation in immune cells underpin persistent cardiovascular risk. Lactylation of histones, a novel modification linking metabolism and epigenetics, exhibits abnormal levels closely associated with adverse remodelling following atherosclerosis and myocardial infarction. Interventions targeting lactate metabolism or specific epigenetic enzymes (such as EP300) hold promise for blocking H3K9la-driven endothelial dysfunction [330]. BET inhibitors (e.g., Apabetalone/RVX-208), acting as epigenetic modulators by targeting BET family proteins (e.g., BRD4), effectively suppress inflammatory gene transcription and regulate lipoprotein metabolism. Clinical trials have demonstrated their potential to reduce major adverse cardiovascular events, potentially representing a novel approach to eradicate metabolic memory via epigenetic pathways. Furthermore, inhibitors targeting methyl transferases such as PRMT3 have demonstrated dual potential in preclinical studies to regulate lipid metabolism and suppress inflammation [331], as shown in Figure 8.

## 7. Conclusions and Perspectives

### 7.1. Core Summary

The pathophysiological mechanisms of CVD extend far beyond mere lipid deposition or haemodynamic impairment, fundamentally representing a dual collapse of metabolic homeostasis and immune equilibrium. This paper systematically elucidates the complete pathogenic chain: ‘glyco-lipid toxicity—immune metabolic reprogramming—cross-organ communication—cardiovascular remodelling’, revealing the central role of metabolic reprogramming in driving cardiovascular immune injury.

Firstly, dysregulation of glycolipid metabolism represents not merely an energy surplus but a priming signal for immune activation. Hyperglycaemia and oxidised lipids, acting as danger-associated molecular patterns (DAMPs), compel immune cells to undergo metabolic reprogramming characterised by enhanced glycolysis and mitochondrial dysfunction through activation of HIF-1α, NLRP3 inflammasomes, and the cGAS-STING pathway [19,23]. This reprogramming not only supplies energy and raw materials for inflammatory responses but also drives epigenetic modifications via metabolic intermediates (such as lactate and acetyl-CoA), forming persistent trained immunity, which underpins systemic metabolic memory [40,44].

Secondly, this pathological process is systemically amplified through cross-organ communication networks. Metabolically impaired liver, adipose tissue, and bone marrow remotely manipulate the microenvironments of vascular walls and myocardium by releasing extracellular vesicles, inflammatory mediators, and metabolic byproducts such as trimethylamine N-oxide (TMAO) [244]. Notably, metabolic reprogramming of haematopoietic stem cells within the bone marrow leads to the sustained output of pro-inflammatory myeloid cells, constituting the root cause of residual cardiovascular risk [248].

Finally, intervention strategies targeting this mechanism demonstrate considerable potential. Drugs such as SGLT2 inhibitors, GLP-1 receptor agonists, and metformin successfully block inflammatory cascades by bidirectionally regulating metabolism and immunity [294,310]. This indicates that only by simultaneously restoring metabolic homeostasis and immune equilibrium can the progression of cardiovascular disease be fundamentally curbed, thereby eliminating residual risks following statin therapy.

### 7.2. Personalised Medicine and Gender Differences

The occurrence, progression, and therapeutic response of cardiovascular and metabolic diseases exhibit marked gender dimorphism, largely attributable to the differential regulation of immune–metabolic networks by sex hormones, genetic background, and environmental factors.

The protective effects of oestrogen: Premenopausal women typically exhibit lower cardiovascular risk than men. Oestradiol has been demonstrated to inhibit macrophage polarisation towards the pro-inflammatory M1 phenotype via the G protein-coupled oestrogen receptor 1 (GPER1), thereby reducing renal fibrosis and inflammatory infiltration [332]. Furthermore, oestrogen reverses male-specific oxidative stress and inflammation in adipose tissue caused by loss of UCP1 function [333].

Sex differences in plaque phenotype: In advanced atherosclerosis, females tend to develop fibrous plaques, which correlates with smooth muscle cell-driven extracellular matrix (ECM) remodelling and activation of the TGF-β signalling pathway, whereas male plaques more frequently exhibit macrophage-mediated inflammation and necrotic core formation [334]. This disparity suggests that therapeutic approaches for women may require greater emphasis on modulating ECM remodelling, whereas men may benefit from enhanced anti-inflammatory strategies.

Metabolic and immune response heterogeneity: In patients with metabolic syndrome and depression, females exhibit heightened inflammatory responses (such as elevated CRP) and a distinct association with gingival bleeding, whilst males are more susceptible to hypertension [335]. In Alzheimer’s disease (AD), the female brain undergoes dramatic neuroimmune and metabolic reprogramming during perimenopause, shifting from glucose metabolism to ketone utilisation, which may explain women’s higher AD risk [336].

Future precision medicine in cardiovascular care should transcend traditional risk factor management and shift towards treatment decisions based on the patient’s individual “immunometabolic phenotype”.

Inflammation-dominant phenotype: For patients with persistently elevated high-sensitivity C-reactive protein (hsCRP) levels (i.e., the “residual inflammation risk” cohort), anti-inflammatory therapies (such as low-dose colchicine, IL-1β or IL-6 inhibitors) may confer significant benefits regardless of lipid profile attainment. Particularly for patients with clonal haematopoietic disorders (CHIP) harbouring TET2 mutations, therapies targeting the NLRP3 inflammasome or its downstream cytokines may prove more effective.

Metabolism-dominant approach: For patients primarily characterised by insulin resistance, obesity, or metabolic syndrome, SGLT2 inhibitors and GLP-1 receptor agonists should be the first-line choice. These agents not only improve metabolic parameters but also exert anti-inflammatory and cardiovascular protective effects by modulating adipokine secretion, inhibiting macrophage foaming, and enhancing mitochondrial function. For specific metabolic defects, such as impaired branched-chain amino acid (BCAA) catabolism, targeted enhancement of catabolic enzyme activity may represent novel therapeutic strategies for sarcopenia and heart failure.

Gender-specific interventions: Gender factors must be fully considered when formulating treatment plans. For instance, in female patients, maintaining balanced oestrogen signalling pathways or employing selective oestrogen receptor modulators may help preserve microvascular function and cognitive health, whereas for males, focusing on androgen-related immunomodulation and metabolic characteristics may prove more critical. Furthermore, personalised monitoring and prevention strategies tailored to gender differences are also required for cardiovascular toxicity arising from cancer treatments [337].

In summary, incorporating gender differences and immunometabolic phenotypes into clinical decision-making frameworks holds promise for achieving precise stratification and personalised treatment of cardiovascular diseases, thereby maximising therapeutic efficacy while minimising adverse reactions.

### 7.3. Challenge

Although targeted immunometabolism offers hope for CVD treatment, clinical translation remains fraught with challenges, centred on precisely regulating the “degree” of immune response.

The balancing act between anti-inflammatory and anti-infective responses: The primary function of the immune system is to defend against pathogens. While the CANTOS trial confirmed the cardiovascular benefits of IL-1β inhibitors, it also revealed the adverse effect of increased risk of fatal infections [307]. This suggests that prolonged, systemic potent immunosuppression may be a double-edged sword. Future strategies should shift towards more precise modulation, such as specifically targeting overactivated metabolic pathways (e.g., inhibiting PKM2 nuclear translocation or blocking NLRP3 assembly), rather than indiscriminately eliminating inflammatory mediators. This approach aims to suppress sterile inflammation while preserving the body’s defence capabilities against infection [324].

Off-target effects of metabolic interventions: Metabolic networks exhibit high interconnectedness and cell specificity. Drugs designed to inhibit glycolysis in macrophages may inadvertently disrupt normal energy metabolism in cardiomyocytes or endothelial cells. Consequently, developing intelligent drug delivery systems with cellular targeting (e.g., via nanocarrier delivery) or microenvironment responsiveness (e.g., ROS-sensitive release) is pivotal for achieving precision therapy [62].

Heterogeneity and individual variation: Significant differences exist in the immunometabolic characteristics of different patients (e.g., gender, genetic background, comorbidities). Identifying the subpopulations most likely to benefit from specific immunometabolic interventions (such as CHIP carriers or those with particular inflammatory phenotypes) through multi-omics technologies within complex clinical contexts represents an essential pathway towards precision medicine [323].

Translational gap between emerging mechanisms and clinical reality: While certain immunometabolic pathways (such as NF-κB activation and oxLDL-mediated foam cell formation) are well-established and pharmacologically targetable, many of the novel pathways discussed herein—including histone lactylation, cGAS-STING signalling, and specific microbial metabolite interactions—are emerging paradigms primarily supported by in vitro and animal models. Bridging the gap from these profound preclinical discoveries to validated human clinical targets remains a significant hurdle, necessitating more large-scale human multi-omics studies to confirm their translational value.

In summary, the theory of cardiovascular immune injury driven by metabolic reprogramming offers a novel perspective for re-examining CVD. Overcoming the aforementioned challenges will propel cardiology towards a paradigm shift from passive defence to active remodelling.

## Figures and Tables

**Table 1:** Metabolic characteristics of distinct immune cell subpopulations and their impact on cardiovascular functional polarisation.

Subset	Metabolic Pathway	Key Regulators	Functional State	Pathological Consequence	PMID
M1 macrophages	Aerobic glycolysis (Warburg effect); PPP	HIF-1α, PKM2	Release of pro-inflammatory factors (IL-1β); impaired phagocytic function	Plaque instability; insulin resistance	[18,26]
M2 macrophages	Fatty acid oxidation (FAO); oxidative phosphorylation (OXPHOS)	PGC-1β, STAT6	Anti-inflammatory; tissue repair; phagocytosis	Promote the resolution of inflammation; stabilise plaques	[18,120]
Th17 cells	Glycolysis; glutamine catabolism	HIF-1α, RORγt	Autoimmune inflammation (IL-17A)	Autoimmune myocarditis; fibrosis	[148,149]
Treg cells	Lipid oxidation (FAO)	FoxP3, AMPK	Immune tolerance; suppression of inflammation	Restrict atherosclerosis; promote myocardial infarction repair	[153,154]
NETosis neutrophil	Glycolysis; oxidative stress (ROS)	NADPH oxidase, PAD4	Release of DNA Nets (NETs)	Immune thrombosis; diabetic cardiomyopathy	[133,143]
CD8+ T cells (terminally exhausted)	Glycolysis (mitochondrial damage)	HIF-1α (persistently stable)	Cytotoxicity; high IFN-γ	Exacerbated myocardial infarction remodelling; intraluminal necrotic core	[158,159]

**Table 3:** Signalling mediators in cross-organ communication and their cardiovascular immunological effects: a summary.

Crosstalk Axis	Mediator	Source	Target	Main Effect	PMID
Heart–fat axis	Fetuin-A	Liver (fat-regulated)	Myocardial/adipose cells	Activation of TLR4-NF-κB induces insulin resistance and inflammation	[240]
Heart–gut axis	TMAO	Gut microbiota metabolism	Vascular/myocardial	Promoting the progression of fibrosis, pulmonary hypertension and heart failure	[261,262]
Heart–intestine axis	Propionate	Gut microbiota (dietary fibre)	T cells/intestinal epithelium	Induces Treg differentiation, inhibits Npc1l1, and reduces plaque formation	[153]
Heart–bone marrow Axis	pEVs (containing miR-499)	Damaged heart/platelets	Haematopoietic stem and progenitor cells (HSPCs)	Promotes emergency bone marrow haematopoiesis and increase pro-inflammatory monocyte output	[255]
Heart–muscle axis	Irisin	Skeletal muscle (exercise-induced)	Macrophages/vascular smooth muscle cells	Inhibits ROS/NLRP3, promote M2 polarisation, stabilise SIRT6	[275,279]

**Table 4:** Ongoing or completed cardiovascular clinical trials targeting inflammation/metabolism and their current status.

Drug	Target	Translation Stage	Key Outcomes	Limitations
Colchicine	Microtubule polymerisation/NLRP3 inflammasome suppression	Approved (FDA approved for ASCVD)	Reduces MACE in chronic coronary syndrome and post-myocardial infarction, independent of statin therapy.	Gastrointestinal side effects are common; benefits in perioperative settings or stroke recurrence remain unclear.
SGLT2 inhibitors	SGLT2/AMPK activation/NLRP3 inhibition	Approved	Reduces heart failure hospitalisation and mortality; improves mitochondrial function and limits macrophage foaming.	Risk of genitourinary infections.
Canakinumab	IL-1 neutralizing antibody	Clinical Stage (Phase III CANTOS)	Reduces MACE risk; significantly greater benefits observed in patients with CHIP (TET2 mutations).	Increased risk of fatal infections; no reduction in all-cause mortality.
Ziltivekimab	IL-6 ligand antagonist	Clinical Stage (Phase II RESCUE)	Significantly reduces hsCRP and thrombotic markers in CKD patients.	Large-scale cardiovascular outcome trial (ZEUS) is currently underway.
Apabetalone (RVX-208)	BET protein (BRD4) epigenetic modulation	Clinical Stage (Phase III BETonMACE)	Modulates lipoproteins and inflammatory genes; reduces MACE risk in specific diabetic subgroups.	The overall trial failed to meet its primary endpoint; requires precise patient stratification.
PKM2 Inhibitors (e.g., Compound 3K)	PKM2-HIF-1α axis inhibition	Experimental/Preclinical	Blocks glycolytic flux; reduces proinflammatory cytokine secretion and myocardial fibrosis.	Potential off-target effects on physiological glucose metabolism; requires further in vivo toxicity profiling.

## Data Availability

The data presented in this study are available on request from the corresponding authors. We confirm that all figures in this manuscript are original content created by the authors using Adobe Illustrator 2020 (Adobe Inc., San Jose, CA, USA). We hold a valid commercial license for this software, which grants full publication rights for all content created with it.
